# Making 3D-Cry Toxin Mutants: Much More Than a Tool of Understanding Toxins Mechanism of Action

**DOI:** 10.3390/toxins12090600

**Published:** 2020-09-16

**Authors:** Susana Vílchez

**Affiliations:** Institute of Biotechnology, Department of Biochemistry and Molecular Biology I, Faculty of Science, University of Granada, 18071 Granada, Spain; svt@ugr.es; Tel.: +34-958-240071

**Keywords:** 3D-Cry toxins, in vitro evolution, rational design, *Bacillus thuringiensis*, toxin enhancement

## Abstract

3D-Cry toxins, produced by the entomopathogenic bacterium *Bacillus thuringiensis*, have been extensively mutated in order to elucidate their elegant and complex mechanism of action necessary to kill susceptible insects. Together with the study of the resistant insects, 3D-Cry toxin mutants represent one of the pillars to understanding how these toxins exert their activity on their host. The principle is simple, if an amino acid is involved and essential in the mechanism of action, when substituted, the activity of the toxin will be diminished. However, some of the constructed 3D-Cry toxin mutants have shown an enhanced activity against their target insects compared to the parental toxins, suggesting that it is possible to produce novel versions of the natural toxins with an improved performance in the laboratory. In this report, all mutants with an enhanced activity obtained by accident in mutagenesis studies, together with all the variants obtained by rational design or by directed mutagenesis, were compiled. A description of the improved mutants was made considering their historical context and the parallel development of the protein engineering techniques that have been used to obtain them. This report demonstrates that artificial 3D-Cry toxins made in laboratories are a real alternative to natural toxins.

## 1. Introduction

Sporulating *Bacillus thurigiensis* produces four non-phylogenetically related insecticidal protein families, the three domain Cry toxins or 3D-Cry toxins, the mosquitocidal Mtx, the binary-like (Bin), and Cyt toxins. All of these proteins form crystals (with the exception of Cry1Ia toxin [1]) concomitantly with the sporulation process. Since the discovery of these crystals in 1953 by Hannay [2], and the demonstration one year later [3] that they were responsible for the already-described entomopathogenic activity of *B. thuringiensis* [4], the study of these toxins has not stopped.

All known Cry toxins (3D-Cry, Mtx like, Bin and Cyt toxins) have been compiled in a brand new database [5], maintained by a commission of experts in charge of assigning a name when a novel Cry protein is described, using the recently proposed structure-based nomenclature rules [6] but with the same basic principles of the rules that were established in 1998 [7]. Most of the 3D-Cry toxins are active against insects from different orders, mainly Lepidoptera, Diptera, Coleoptera, Hemiptera (low toxicity for some aphids), and Hymenoptera, but some of them have other targets such as nematodes, snails, and even cancer cells [8]. Recently, a toxin active against Orthopteran insects has been described [9].

3D-Cry toxins are the best-characterized among the Cry proteins. Among them, Lepidoptera-active toxins are the best known from a mechanistical point of view. 3D-Cry toxins, synthesized as inactive protoxins by *B. thuringiensis*, have to undergo a proteolytic activation process in the guts of the susceptible insects to become active. Since the elucidation of the first three-dimensional structure of the active part of the Cry3A toxin by Li et al. in 1991 [10], the structure of nine other members of the family have been elucidated (Cry1Aa [11], Cry3Bb1 [12], Cry1Ac [13], Cry2Aa [14], Cry4Ba [15], Cry4Aa [16], Cry8Ea1 [17], Cry5B [18], and Cry7Ca1 [9]). Recently, the 3D structure of the 120 KDa Cry1Ac1 prototoxin has also been described [19], showing seven different domains (DI–DVII). Although very different at the amino acid sequence level, the structural disposition of 3D-toxins is very conserved. Active toxins present three very distinct structural domains (hence their name of 3D-toxins), each of them with a specific function. Domain I, at the N terminus end of the protein, is comprised of a bundle of seven α-helices and is responsible for the formation of a pore in the midgut cells of susceptible insects. Domain II, the middle domain, formed by three antiparallel β-sheets, plays an important role in receptor recognition. Domain III, a two antiparallel β-sheet sandwich, is thought to be involved in receptor binding and pore formation [20]. Protoxin Domains IV and VI are α-bundles similar to domains present in other proteins such as spectrin or the bacterial fibrinogen-binding complement inhibitor. On the other hand, Domains V and VII are β-rolls resembling carbohydrate-binding proteins such as sugar hydrolases [19]. Although the functions of DIV–DVII are not known, they have been related with crystal formation, toxin stability, and selective solubilization in the insect gut. In addition, it has recently been suggested that Domains V and VII could also be interacting domains with proteins present in gut membranes, and hence be involved in the recognition of receptors of the full toxin [21].

3D-Cry toxins have been exploited in insect pest management since the late 1930s [22] in agriculture and against health-related insect populations. 3D-Cry toxins have been used in agriculture, not only as spray formulations, but also in plant transgenesis to protect plants such as maize, cotton, soybean, potato, and tomato from insects [23]. 3D-Cry toxins are extremely efficient and their main characteristic is the narrow spectrum of action that each toxin shows. Their specificity is due to a complex mechanism of action, and although it is not completely understood and many questions remain unanswered, it is known to involve several steps. Currently, two models to explain the mechanism of 3D-Cry toxins have been proposed: the sequential binding model and the signaling pathway model. The sequential binding model involves crystal solubilization at a specific pH, proteolytic activation by gut digestive enzymes, receptor recognition at the membrane cells of susceptible insects, helix 1 proteolysis, conformational changes of the molecule, polymerization, and finally, membrane insertion. The signaling pathway model shares the steps of crystal solubilization, proteolytic activation, and receptor binding, but death of the cell is not explained by toxin insertion in the membrane but by the activation of cell apoptosis. Both models [22] have in common the need of 3D-Cry toxins to bind to a specific receptor at the enterocytes and possibly many other interacting receptors are required for the toxin mode of action. Among them, aminopeptidase N (APN), cadherin-like proteins (BT-R1, BtR175, HevCaLP), alkaline phosphatase (ALP), and ABC transporters have been described, with the first two receptors being the best characterized.

Everything that we know today about the mechanism of action of 3D-toxins has been mainly obtained from two sources of information: the construction of 3D-Cry mutants and the study of the resistance phenomena that insects have shown to the action of 3D-Cry toxins [24,25]. The study of mutant proteins is one of the pillars for the elucidation of any mechanism of action of any protein. The principle is simple: if one amino acid of the protein is essential for its mechanism (or the structure), when changed for another amino acid, the functionality of the protein is modified. This is a consequence of the biological principle that the structure and function of any protein are always linked. In the case of 3D-Cry toxins, thousands of mutants have been constructed to elucidate which amino acids are important for maintaining the three-dimensional structure of 3D-toxins, which are responsible for the binding to their receptors, and which are relevant for the solubilization or activation of the toxins. The information provided by the behavior of these mutants has allowed researchers to propose models that explain the mechanism of action of these natural machines, specialized in killing insects.

However, constructing mutant proteins also presents the possibility of obtaining functional variant proteins with different behaviors, even with improved activity. By manipulating the DNA sequence that codifies the 3D-toxins, versions of toxins completely novel in nature with an enhanced activity toward a particular insect, with a broader insect target, or with a novel activity against a non-susceptible insect can be obtained. The more we understand 3D-Cry toxins, the more creative we can be in the generation of artificial toxins. The fact that a high number of molecular techniques for DNA manipulation is available ensures that in the generation of 3D-Cry toxins variants, only our imagination is the limit.

The techniques available for DNA manipulation and generation of novel mutants or variants can be separated in two main groups: (i) those where mutations are generated randomly and afterward, there is a screening process and the desired mutant is selected, and (ii) those where there is a rational design behind the mutant construction. A deep understanding of protein structure and function is needed in order to use rational design, as we must decide which amino acid (or amino acids) is (or are) going to be changed and which amino acid is going to be substituted for. In contrast, random mutation can be generated anywhere in the protein sequence without previous knowledge, and selection of the suitable variant is carried out later on. The latest group of techniques is known as directed evolution or in vitro evolution of proteins in protein engineering. It is a process that simulates natural evolution, introducing a mutation and selecting it if it represents an advantage, but carried out in a laboratory context and with human intervention. For this review, I would like to use the term “in vitro evolution” in a much broader sense. I would like to include those mutants obtained by rational design and those obtained when investigating the function of the toxin, but instead of getting an impaired mutant with no function, a better 3D-Cry toxin is generated. If the reader grants me this license, then the purpose of this review makes much more sense as the intention is to compile all of the mutants generated in 3D-Cry toxins, independently of the objective of the work and the technique used. These man-made (and woman-made) mutants presented here collectively represent the “in vitro evolution” that 3D-Cry toxins have experienced thanks to the work of hundreds of researchers worldwide in a relatively short period of time. Although it must sound highly pretentious comparing human work that of Mother Nature, the fact is that some of the mutants obtained in a laboratory sometimes show better characteristics than natural toxins, at least from a practical point of view.

Previous reports have reviewed the enhancement of 3D-Cry toxin activity [26,27], but this time I would like to give a historical perspective of what the methodological context of protein engineering was like when the enhanced toxins were obtained. Therefore, apart from the objective of updating the information to the present time, this review has the purpose of describing the parallel development of molecular techniques that were used for constructing the improved versions of 3D-Cry toxins. The mutants reviewed here represent all successful variations of the 3D-Cry toxins that have been experienced in a laboratory, collectively, through many different techniques, even if the intention of the researchers was not to obtain an improved mutant. In this report are compiled all the enhanced mutants constructed through the history of 3D-Cry toxin research (Table 1; shown at the end of section two), together with all the relevant positions in the toxin molecules. The sequences of all these mutants are detailed in Table S1. I believe that this is a valuable source of information that I hope will contribute to the production of even better molecules in the future. 

## 2. “In Vitro Evolution” of 3D-Cry Toxins: An Historical Perspective

Although Cry toxins are very efficient molecules and only very minute quantities are required for toxicity, the obsession to improve their efficiency has led to the development of diverse strategies [28]. The developed strategies include (i) the combination of several Cry toxins to increase efficiency toward a specific target [29]; (ii) co-expression with other *B. thuringiensis* proteins such as the P20 protein to provide protection in the larval gut environment [29,30], or chitinases for peritrophic membrane degradation [31]; (iii) combination with chemical compounds such as calcofluor for peritrophic membrane digestion [32], or coating with Mg(OH)_2_ to increase their resistance to UV light [33]; (iv) combination with other insecticidal toxins such as Cyt toxins [34,35,36,37], VIP toxins [38], Bin toxins [39], Metalloproteinase Bmp1 [40], or insect-specific scorpion toxins [41] that synergize their effect; (v) combination with insect chaperones such as Hsp90 chaperone [42]; or (vi) expression of Cry toxins in other backgrounds such as baculovirus [43]. Other strategies developed to increase potency include the fusion of 3D-Cry to other toxins such as neurotoxins (Huwentoxin-I [44], ω-ACTX-Hv1a [45], or huwentoxin XI [46]) present in spiders venom, VIP proteins [47], the N-terminal region of PirB toxin from *Photorhabdus luminescens* [48], or the fusion to other proteins that provide interesting domains such as garlic lectin [49,50], cellulase-binding peptides [51], or *Escherichia coli* maltose binding protein (MBP) [52], thus rendering chimeras with improved activity. Lately, the combination or co-expression of Cry toxins with peptides with sequences similar to natural receptors, or other proteins present in the gut cells, is attracting the attention of researchers. Among them, peptides such as HcAPN3E, derived from an APN receptor in *Hyphantria cunea* [53], or cadhering fragments [54,55,56,57,58,59] can be mentioned.

Some of the mechanisms for enhancing 3D-Cry toxin toxicity above-mentioned are common strategies in the field of protein engineering (like the fusion of 3D-Cry toxin with other proteins), but are out of the scope of this review as the intention here is to describe specific changes carried out in the amino acid sequence of 3D-Cry toxins that are responsible for the improvement of toxicity.

The starting point in the history of the 3D-Cry toxin “in vitro evolution” could be set at the beginning of the 1980s. At that time, researchers started to understand that the differences in activity of the different *B. thuringiensis* isolated strains were due to the expression of different Cry toxin variants. In 1981, the first *cry* gene was cloned and expressed in a heterologous system [60], a milestone that represented the beginning of molecular biology for Cry toxins. A few years later, the sequence of the first *cry* gene and its deduced amino acid sequence were determined [61]. When the sequence of several other 3D-Cry toxins was available, researchers realized that areas with conserved and variable sequences were present in all of the Cry toxins, so conserved and variable blocks were established. Pretty soon, “variable regions” were related to the specificity observed in 3D-Cry toxins and represented a good starting point for the manipulation of the molecules in order to increase the activity toward insects or expand their target insects. In this sense, the patent number EP0228838 was filed in 1986 by the Mycogen Corporation at the European Patent Office [62] to commercially protect the idea that activity of 3D-Cry toxins could be modified and improved by exchanging specific “variable” regions at their sequence, and a novel method with which to do it. Since then, the history of Cry toxin “in vitro evolution” has not stopped and continues until the present day. Cry toxin history is long and exciting, and has been possible thanks to the development of the molecular tools for DNA manipulation. A description of all the molecular techniques used for the improvement of 3D-Cry toxins will also be made.

### 2.1. Evolution by Chemical Mutagenesis and Homologue Scanning Mutagenesis, the First Molecular Techniques Used for Cry-Toxins “In Vitro Evolution”

In the 80s, tools for molecular biology were extremely limited. To provide the reader with some background context, restriction enzymes, essential for DNA manipulation nowadays, had only just been recently discovered [63] and the number available was very low. The Sanger method for determining DNA nucleotide sequences had just been developed [64,65] and chemical mutagenesis was pretty much the only tool available for the “in vitro evolution” of Cry toxins. The technique consisted in subjecting *cry* genes to the action of mutagenic substances such as bisulfite or formic acid to obtain random mutations. Bisulfite, a single DNA strand mutagen, converts cytosines into uracils by deamination [66], rendering a transition from cytosine to thiamine or guanine to adenine, depending on the mutagenized strand (sense or antisense strand). Formic acid depurinates DNA by hydrolyzing the N-glycosyl bond between the ribose and purines [67], and when polymerization takes place using this mutated DNA as a template, a transversion is produced.

Chemical mutagenesis has been used to improve the activity of 3D-Cry toxins [68], specifically on CryIA(b) (the original names of the Cry toxins are maintained in this review). After cloning the *cry1A(b)* gene in the M13 phage, in order to obtain single stranded DNA, and setting mutagenesis conditions to obtain 2–3 mutations per gene (by limiting the exposure time of the DNA to the mutagen), the authors obtained eleven mutant toxins that were 3–5 times more toxic toward *Heliothis virescens* than the parental toxin. When the DNA sequence of these clones was determined, a wide range of substitutions was observed, all of them at Domain I of the toxin, although the distribution in the molecule was not known at that time, as none of the 3D-Cry toxin three-dimensional structure had been elucidated yet.

Another option that researchers had available at that time was the technique called homologue-scanning mutagenesis. The technique consisted in exchanging equivalent regions of two 3D-Cry toxins in order to create hybrid molecules. The fragments exchanged were limited by the restriction enzymes that these *cry* genes had in common. Without a doubt, these kinds of exchanges would have been done by PCR nowadays, but this technique was not developed until 1988 [69], and it took time until the research community understood its potential in protein engineering. The main objective of using homologue-scanning mutagenesis was to understand the remarkable specificity that Cry toxins had against the same insects (at the beginning most of them Lepidoptera), but soon researchers understood the potential that this knowledge also had from a practical point of view; identifying regions responsible for the specificity was of paramount importance as they could be manipulated and modified to extend or change the specificity toward other insects.

One of the firsts reports using homolog-scanning mutagenesis in a Cry toxin was published in 1989 [70]. Here, the authors used two *cry* genes, called at the time *icpA1* and *icpC73*, as the first systematic nomenclature for Cry toxins had not been implemented [71] (in fact, the first proposed nomenclature was published the same month as this report). The authors exchanged several fragments between ICPA1, which is highly toxic toward *Bombyx mori*, and the non-toxic ICPC73 by making use of the restriction sites that these two *cry* genes have in common. After the resulting novel toxins were bioassayed, the authors observed that when conserved blocks were exchanged, the activity of the hybrid toxins did not change. However, when variable regions were exchanged, the activity of the toxins dramatically changed and could be redirected toward other insects. In other words, ICPC73 became toxic toward *B. mori* when certain regions from ICPA1 were substituted in its sequence. The region responsible for *B. mori* specificity in the ICPA1 toxin was even narrowed down to the region between residues 332 and 450, a region that we know today includes loop 1, loop 2, and loop 3 in most of the 3D-Cry toxins and has been proven to be involved in receptor recognition and specificity. These authors suggested that if these regions were indeed responsible for specificity in other “IPC” toxins, that they would be an excellent area for mutation in order to redirect activity toward other insects.

The same strategy and the same toxins (now called CryIA(a) and CryIA(c)) were tested in other insects by Schnepf et al. [72]. Both toxins showed a similar activity against *Manduca sexta*, but very different activities against *H. virescens* (as CryIA(c) is 50 times more potent than CryIA(a) for this insect). With these models, the specificity determinant regions for Lepidoptera were determined and it was demonstrated that Cry toxins could be “in vitro evolved” and their specificity could be changed completely. The same year, the specificity-determining region for a Dipteran toxin was determined [73] using homologue-scanning mutagenesis with the CryIIA toxin (active against Diptera and Lepidoptera) and CryIIB (active against Lepidoptera only). They determined that when a 241 nt segment from *cryIIA* was inserted on the *cryIIB* gene, the lepidopteran toxin showed a broader insect spectrum, also becoming active against Diptera. They even narrowed down the region responsible for the specificity of CryIIA protein toward mosquitoes to 76 amino acids.

A following work [74], done by the pioneers in the use of homologue-scanning mutagenesis in a 3D-Cry toxin, demonstrated the same effect in the CryIA(c) toxin and two other economically important pests (*H. virescens* and *Trichoplusia ni*). They defined the minimal region responsible for the toxicity of CryIA(c) as the region between amino acid 332 and 450, an equivalent region described in CryIA(a). Surprisingly, one of the hybrids obtained (hybrid 4109) showed an enhanced activity compared to the parental toxins, being 30 times more toxic than the most potent natural toxin known for *H. virescens*. This represented the first proof that by changing specific areas in the sequence, not only was it a means of modifying the specificity of the 3D-Cry toxins, but also a way of increasing their activity.

At this point in 3D-Cry toxin history, researchers started to have the overall view that 3D-Cry toxins were modular structures and that their function could easily be manipulated by exchanging parts of the molecules.

### 2.2. Evolution by Domain Swapping

With the elucidation of the first three-dimensional structure of a Cry toxin (Cry3A; PDB: 1DLC) by Li et al. [10], researchers had the opportunity to “see” the spatial distribution of amino acids in a 3D-Cry molecule and to verify that active 3D-Cry toxins showed three very well defined domains (hence their name). Through comparison with other proteins, hypothetical functions for some domains were described. For example, Domain I, with seven long α-helices, long enough to span the lipid bilayer of the cell the membrane, was associated with the pore formation function. Domain II, with three loops at the apical part showing highly variable amino acid sequences, was suggested to be responsible for specificity.

Once the structure of Cry toxins was elucidated and the concept that each domain had a function was established, the idea of improving and redirecting the toxicity of a Cry toxin by exchanging complete domains was reinforced. Many reports of what was called domain swapping were produced using molecular strategies such as in vivo intramolecular recombination, cloning, or overlapping PCR.

**Figure 1:**
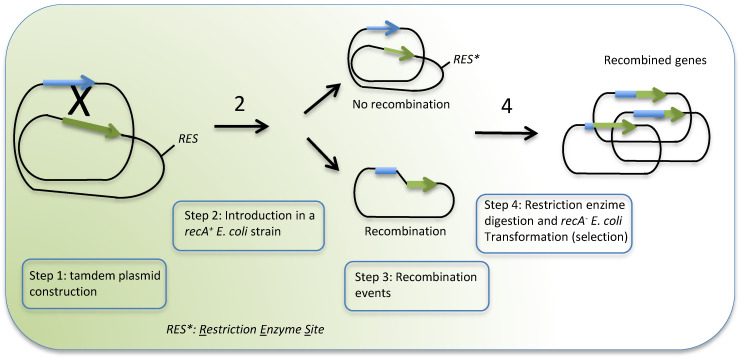
In vivo intramolecular recombination. A tandem plasmid is constructed by the cloning of two truncated genes, one truncated at the 3′ end (blue gene) and the other one at the 5′ end (green gene), leaving an overlapping region were recombination is desired. The plasmid contains a restriction site to further discriminate if recombination took place. Plasmid is then introduced in a *recA*^+^ *E. coli* strain and DNA recombination is allowed. After plasmid extraction from the *E. coli recA*^+^ strain, the pool of plasmids are digested with a restriction enzyme and selected in a *recA*^-^ *E. coli* strain. Each clone represents a recombination event where the two toxins genes have been fused [77].

The technique known as in vivo intramolecular recombination [75] is based on the construction of a “tandem plasmid” where two truncated proteins, in direct repeated orientations, are cloned (Figure 1). The truncated genes (one lacking the 3′ end of the gene and the other the 5′ end) only overlap in an area where the recombination is intended. Tandem plasmid contains an enzyme restriction site to further discriminate between recombinant plasmids (where the restriction site is lost) and the parental one. Once the tandem plasmid is introduced in a recombinase positive *E. coli* strain (Rec^+^), random recombination takes place at the homologue regions and novel hybrids or chimeras are produced.

One of the first works using in vivo intramolecular recombination to obtain 3D-Cry toxin hybrids was reported by Caramori et al. [76]. They cloned two truncated toxin genes (for CryIA(a) and CryIA(c)) with overlapping variable regions (63% identity). After the hybrid toxins were bioassayed against several Lepidoptera, it was determined that hybrid 32 (pHy32) and hybrid 45 (pHy45) were more active toward *T. ni* and *Heliotis* sp., respectively, than any of the parental toxins. Two of the hybrid toxins (pHy104 and pHy122) with the same amino acidic sequence even gained a novel activity against *Spodoptera littoralis* as none of the parental toxins were active against this insect.

In vivo intramolecular recombination has been used for the combination of other 3D-Cry toxins and some of them rendered enhanced toxins. This is the case for the combination of CryIC and CryIE toxins [77], both active against Lepidoptera, but with different specificities. CryIC is particularly active against *Spodoptera exigua* (LC_50_ 26 ng/cm^2^) and *Mamestra brassicae* (LC_50_ 8 ng/cm^2^) while CryIE is not (LC_50_ > 1000 ng/cm^2^). The authors constructed two tandem plasmids with truncated genes overlapping at Domain II and III of the toxins to construct CryIC-CryIE and CryIE-CryIC hybrid proteins. One of the resulting hybrids, named G27 and containing Domain I and II from CryIE and Domain III from CryIC, was toxic to *S. exigua* (LC_50_ 2 ng/cm^2^). The reverse hybrid (DI and DII from CryIC and DIII from CryIE) was not toxic at all, meaning that the CryIC Domain III was involved in the specificity against this insect.

Using in vivo intramolecular recombination, de Maagd et al. [78] demonstrated that the moderate toxicity of CryIA(b) toward *S. exigua* could be enhanced by constructing a hybrid between DI and DII from CryIA(b) and DIII from a highly active toxin (CryIC). Hybrid H04 showed an increase in toxicity of more than 66-fold (CL_50_ from > 100 μg of toxin/g of diet to 1.66 μg/g) compared to Cry1Ab. The activity of the novel hybrid was even better than the parental CryIC toxin (LC_50_ 11 μg/g), showing a 6.6-fold increase in toxicity. The authors also determined that binding sites of CryIA(b) and CryIC to *S. exigua* Brush Border Membrane Vesicles (BBMVs) were different for both toxins and argued that in the case of insect resistance, hybrid H04 could be of great use.

Other hybrid toxins obtained by in vivo recombination from the less studied Cry1Ba, Cry1Da, and Cry1Fa toxins (Cry toxins nomenclature updated following [7] rules) were reported to have an improved activity [79]. This was the case of hybrid BBC13 (DI-DII from Cry1Ba and DIII from Cry1Ca) that showed an 11.8-fold increase in toxicity toward *M. sexta*, or hybrid BBC15 (DI-DII of Cry1Ba and DIII of Cry1Ca), which showed 8.3-fold and 7.8-fold increases in toxicity against *S. exigua* and *M. sexta*, respectively, or hybrid FFC1 (DI-DII from Cry1Fa and DIII from Cry1Ca) that showed a 5.5-fold increase toward *S. exigua*.

The latest example of hybrids made by in vivo recombination were made from Cry1Ca and Cry1Fb toxins, and DIII from Cry1Ac [80]. Hybrids RK15 (Cry1Ca/Cry1Ca/Cry1Ac) and RK12 (Cry1Fb/Cry1Fb/Cry1Ac) showed an increase in activity, compared to wild type toxins Cry1Ca and Cry1Fb, of more than 172 and 69 times toward *H. virescens*, respectively.

Another way of performing domain swapping and constructing hybrid toxins has been through standard cloning using restriction enzymes (already present or expressly created). This is the case of the hybrid Cry1C/Ab [81], constructed with the first 2194 nucleotides from *cry1C* (731 aa) and the last 1295 nucleotides (432 aa) of the 3′ end of the *cry1Ab* gene. The fusion was possible thanks to the presence of a unique *Kpn*I site in a conserved region of the two genes. The Cry1C/Ab hybrid was 3, 4, and 35 times more active against *S. littoralis*, *Ostrinia nubilalis*, and *Plutella xylostella*, respectively, than the parental Cry1C toxin.

Domain swapping of Coleoptera active toxins has also been successfully accomplished by standard cloning [82], although in this case, it was necessary to introduce restriction sites (*Rsr*II) to obtain the hybrids. Hybrid 1Ia/1Ia/1Ba (DI-DII from Cry1Ia and DIII from Cry1Ba; LC_50_ 22.4 μg/mL) was 2.5 and 7.5 times more toxic than their parental toxins, respectively. Hybrid 1Ba/1Ia/1Ba (DI from Cry1Ba, DII from Cry1Ia, and DIII from Cry1Ba, LC_50_ 7.94 μg/mL) increased its activity even further, showing 17.9 times more potency than the parental Cry1Ba toxin. The latest hybrid toxin was almost as toxic as the Cry3Aa toxin, the most active natural protein for the Colorado potato beetle.

More recently, and thanks to the determination of the Cry1Ac1 full-length toxin 3D structure ([19]; PDB 4W8J), Zghal et al. [21] constructed a 116 KDa chimeric toxin called Cry(4Ba-1Ac) by fusing DI–DIV from Cry4Ba to DV–DVII from Cry1Ac1, using PCR amplification and cloning techniques. This represents a unique case in which other domains, apart from Cry toxin toxic domains, have been swapped. The chimeric toxin showed low toxicity toward *Culex pipiens* when expressed in an acrystalipherous *B. thuringiensis* strain (HD1 CryBpHcry(4Ba-1Ac)), but when co-expressed in a Cry2Aa producing strain (BNS3pHTcry4BLB), the activity increased from 10% mortality to 100% mortality at 200 μg/L. The LC_50_ of the strain bearing only Cry2Aa switched from >>200 μg/L to 0.84 μg/L when co-expressed with the chimeric toxin, meaning that an increase in toxicity of more than 238-fold was produced. This synergy was also observed when Cry2Aa and the chimeric toxin Cry(4Ba-1Ac) were bioassayed in combination. The authors suggested that the increased toxicity could be explained by a better solubilization of the crystals and also proved the importance of the protoxin domains (DIV–DVII) in the stability and the activity of the Cry toxins, a fact that will possibly be exploited in the future.

3D-Cry toxin domain swapping has also been obtained by overlapping PCR [83]. In this case, a hybrid using toxins from different classes, one coleopteran (mCry3A) and one lepidopteran specific (Cry1Ab), was obtained. DNA regions codifying for DI and DII from mCry3A [84] and DIII from Cry1Ab were amplified, containing an overlapping region at the 3′ and 5′ ends, respectively. Amplicons were used as a template in an overlapping PCR with flanking primers. The resulting chimera (called eCry3.1Ab; GenBank GU327680) was highly active (93% mortality) against *Diabrotica virgifera virgifera* when bioassayed with a toxin concentration ranging from 5 to 10 μg of toxin per mL of diet. Although the authors did not determine the LC_50_ of eCry3.1Ab, the toxicity observed was much higher than the previously reported parental mCry3A toxin (LC_50_ 65 μg/mL; [84]).

A more recent example of constructing improved mutants by domain swapping using overlapping PCR [85] was the hybrid toxin Cry1Ac-Cry9Aa containing DI from Cry1Ac and DII and DIII from Cry9Aa. The hybrid showed 4.9 times more activity against *Helicoverpa armigera* than the wild type Cry9Aa. In addition, a Cry1Ac-Cry9AaMod toxin, where helix 1 was proteolytically removed, showed a 5.1-fold increase in toxicity.

### 2.3. Evolution by Site-Directed Mutagenesis

Site directed mutagenesis is a molecular strategy used to create specific changes in the amino acid sequence of a protein in order to evaluate its role in the molecule. If an amino acid is involved in the mechanism of action of a protein, when changed, the function of the protein is modified and normally abolished. However, the technique could also be used as a means of obtaining novel proteins with improved characteristics.

Site-directed mutagenesis consists of the in vitro synthesis of the codifying DNA of a protein in which one or several nucleotides are changed in a specific site in order to produce a mutant protein. The changed nucleotide is normally introduced using a mutant primer that is in vitro extended thanks to a DNA polymerase (the *E. coli* DNA polymerase Klenow fragment in the early days, and *Taq* polymerase lately when PCR [69] and site-directed mutagenesis by PCR [86] were developed). The in vitro synthesized DNA (mutant DNA) is counter selected from the wild-type DNA, and the mutant protein is expressed and tested for activity.

The elucidation of the three-dimensional structure of a 3D-Cry toxin facilitated the design of mutants for site-directed mutagenesis as the position of the amino acid to be changed was localized in the space together with its interactions with other amino acids in the molecule. Hundreds of site-directed mutagenesis studies in many Cry toxins have been performed, allowing the elucidation of the function of each domain of the protein. Although most of the mutants obtained by site-directed mutagenesis showed an impaired or diminished toxicity, in some cases, the activity of the resulting mutants was higher than the parental toxins. These mutants were not as useful as the impaired mutant to elucidate the mechanism of action of 3D-Cry toxins, but served to settle the concept that activity improvement was possible with only the change of a single amino acid.

This was the case in the work reported by Wu et al. [87] in which the authors constructed 31 mutants at two conserved regions at CryIA(c) Domain I (residues from 84 to 93 and from 160 to 177). Although most of the mutant toxins showed no toxicity, or no change in toxicity at all, one of them, the mutant H168R, localized at the hydrophobic face of the amphypatic α-helix 5, showed a 3–5-fold increase in toxicity toward *M. sexta* compared to the wild type toxin. Another example of Domain I improved mutants was obtained by investigating the role of nine tryptophan residues in the toxicity of Cry1Ab toward *M. sexta* [88]. These authors found that a conservative change to phenylalanine (W73F, W210F, W219F) produced mutant toxins 3.3, 1.5, and 2.3 times more toxic than the parental toxin. In a similar study [89], two α-helix 5 Cry1Ab mutants (V171C and L157C) with a 25-fold and 4-fold increase in toxicity toward *Lymantria dispar*, respectively, were reported.

Site directed mutagenesis studies carried out in 3D-Cry toxins showed that Domain II was particularly sensitive to amino acid changes [90,91]. Some of the mutations performed in this domain were very successful in improving the toxicity against certain insects, and represented an excellent place for redesigning the activity of 3D-Cry toxins. This was the case reported by Rajamohan in 1996 [92] where two single mutants N372A, N372G, and a triple mutant DF-1 (N372A, A282G, L283S) of the Cry1Ab toxin were constructed by site-directed mutagenesis. These residues were localized between the Cry1Ab α-helix 8a and α-helix 8b at Domain II. When bioassayed, N372A and N372G were, respectively, 8.5 and 8.3 times more potent for neonates of *L. dispar* than the parental Cry1Ab toxin, and 9.61 and 9.51 times more potent for fourth instar larvae, respectively. The DF-1 mutant showed an increase in activity of 36- and 17-fold toward neonates, and fourth instar larvae, respectively. The triple mutant was even more toxic (4-fold) than Cry1Aa, the most potent natural toxin active against *L. dispar*.

Loop 1 from Domain II has also been a successful place for mutagenesis, rendering improved mutants in the coleopteran active Cry3A toxin [93]. Site-directed mutagenesis at positions R345, Y350, and Y351 rendered eight single mutants, four double mutants, and three triple mutants, two of them showing an enhanced activity against *Tenebrio molitor*. Mutant A1 (R345A, Y350F, Y351F) and mutant A2 (R345A, ΔY350, ΔY351) were 11.4 and 2.7 times more active than wild type Cry3A, respectively. In addition, an enhanced activity of these two mutants against two other Coleoptera species, *Leptinotarsa decemlineata* and *Chrysomela scripta*, was also observed. Although changes introduced in these mutants were not very drastic (as Y and F differ only by an OH group), differences in toxicity were remarkable. In addition, loop 3 of the Cry3A toxin also proved to be relevant for toxicity enhancement [91]. The triple mutant S484A, R485A, G486A, showed a 2.4-fold increase in toxicity toward *T. molitor* compared to the wild type toxin.

Another place in Domain II where the substitutions introduced by site-directed mutagenesis rendered an enhanced toxin was the area known as D block or Dipteran specific block [73] in the dual toxin Cry2A (dual as it is also active against Lepidoptera). Cry2Ab is not a very potent toxin toward *Anopheles gambiae* (LC_50_ 540 ng/mL), but when residues from 307–337 were mutated, three mutants (N309S, F311I, and A334S) with enhanced toxicity (1.17, 3.17, and 6.75-fold, respectively) were obtained [94]. One of the mutants, A334S, was even more toxic than the highly active natural toxin Cry2Aa [95].

Recently, 3D-Cry toxin Domain III has also been targeted for mutation by site-directed mutagenesis, and mutants with improved characteristics have been found. This is the case of the work reported by Lv et al. [96] in Cry1Ac5. Although the structure of this 3D-Cry toxin is not elucidated, 3D structure modeling using known structures (Cry1Aa (PDB:1CIY), Cry2Aa (PDB:1I5P), Cry3Aa (PDB:1DLC), Cry3Bb1 (PDB:1JI6), and Cry4Ba (PDB:1W99) was useful in localizing the loop sequence between β-20 and β-21 (^576^NFTSSLGNIV^586^). Two mutants obtained by site directed mutagenesis, S581A and I585A, showed 1.72- and 1.89-fold increases in toxicity toward *H. armigera*.

Another area that has recently been demonstrated to be susceptible to improvement by site directed mutagenesis is the β sheet 16 in Domain III. A study of alanine substitution in Cry1Ab [97] performed on this area rendered mutants S509A, V513A, and N514A, which showed an increase in toxicity toward *Spodoptera frugiperda* of more than 9.5-, 12.7-, and 51-fold, respectively. As N514 was the most relevant position experiencing toxicity enhancement in β-16, saturation mutagenesis was performed at this position (N was changed for any of the other 19 amino acids). Some of the obtained mutants, N514F, N514H, N514K, N514L, N514Q, N514S, and N514V showed a 44-, 16-, 7-, 9-, 26-, 23-, and 9-fold increase in toxicity, respectively, when compared to the wild type. An equivalent mutation in Cry1Fa (N504A), a more potent toxin than Cry1Ab for *S. frugiperda*, rendered a mutant 6–11 times more toxic than the wild type Cry1Fa in different populations of *S. frugiperda* from different countries. The authors suggested that the increase in toxicity correlated with an increase in the stability of the mutants toward gut proteases and an increase in BBMV binding.

The fact that two other mutants in β-16, this time in Cry1C [98], showed a slight increase in toxicity compared to the wild type (mutant V509A was 1.6 times more toxic than Cry1C for *M. sexta* and mutant N510A was 1.5 times more toxic toward *S. frugiperda*) suggest that β-16 is a recently discovered site for toxicity improvement.

The loop sequence between β-18 and β-19 in Domain III also seems to be a relevant region for toxicity enhancement. Mutant N546A [99] showed a slight increase in toxicity (1.8-fold) in Cry1Ac toxin, which correlated with a binding increase toward *H. armigera* BBMVs [100].

Single mutations in Domain III have also been described to be useful for toxicity enhancement. This was demonstrated in the nematocidal toxin Cry5Ba [101]. Investigating the role of the 3 asparagines present in block 3 of the toxin found that alanine substitution, by site directed mutagenesis, rendered a mutant (N586A) with a 9-fold increase in toxicity (GIC_50_ from 42.11 to 4.75 ng/mL) toward *Caenorhabditis elegans*. Mutant N586A was surprisingly soluble in a wide range of pHs (from pH 5 to pH 12), which correlated with the observed increase in toxicity.

### 2.4. Evolution by Rational Design

Rational design is a particular case of site-directed mutagenesis performed only when the knowledge of the structure and the function of the protein under study are very deep. In rational design, a hypothesis is formulated and proven by the construction of mutants with single amino acids changes, deletions, or insertions, normally performed by site-directed mutagenesis. Several works have described successful evolution of 3D-Cry toxins by rational design, although some of these were found by accident trying to prove a different hypothesis. This was the case in the work reported by Angsuthanasombat et al. in 1993 [102] where the authors were interested in demonstrating that R203 in Cry4B toxin was essential for proteolytic processing and the α-helices mobility of Domain I. For that, they replaced the R (proteolytic site) with an A, expecting that toxicity would be completely lost as a consequence of the impossibility of the helices to move properly. The effect was completely the opposite as the R203A mutant was 2.8 times more toxic to *Aedes aegypti* than the wild type.

An example of rational design where the researcher did prove their hypothesis was that reported for the evolution of Cry4Ba toxicity [103]. The Cry4Aa toxin is highly active against four species of mosquito (*Ae. aegypti*, *Anopheles quadrimaculatus*, *Culex quinquefasciatus*, and *C. pipiens*), however, the closely related Cry4Ba toxin (the one to be evolved) showed toxicity toward *Ae. aegypti* and *An. quadrimaculatus*, but not to *C. quinquefasciatus* and *C. pipiens*. Through site-directed mutagenesis, the authors delimited the putative loop 3 in Cry4Ba (VIDYNS) and in Cry4Aa (IPATYK), as the Cry4Ba and Cry4Aa 3D structures were not determined until 2005 and 2006, respectively [15,16]. The authors mutated the Cry4Ba loop 3 by replacing D454 with a P and inserting AT after position 454 to yield a novel toxin (named 4BL3PAT) with a novel loop 3 sequence (VIPATYNS). This small change increased Cry4Ba activity 700-fold toward *C. quinquefasciatus*, and by 285-fold to *C. pipiens*. Other versions of the novel toxin were also constructed in loop 3 by substitution of the PAT motive to other motives (4BL3AAT, 4BL3GAT, 4BL3GAV, 4BL3PAA, and 4BL3AAA), showing an activity gain toward *C. quinquefasciatus* and *C. pipiens* in almost all variants (with the exception of 4BL3AAA toward *C. quinquefasctiatus*). The study of the mechanism of the 4BL3PAT mutant, compared to the wild type toxin, showed that both toxins had little difference in the ability of reversible binding to *Culex* BBMVs, a similar capability of irreversible binding, but 4BL3PAT showed a higher pore-forming ability than the Cry4Ba parental toxin. The authors identified two novel proteins in the BBMVs, which are 35 and 36 KDa in size, that the novel mutant bonds to instead of the parental toxin, proposing that these two proteins could be functional receptors and explain why the 4BL3PAT variant is toxic to the *Culex* species (although it was not proven). One year later [104], the same authors reported the evolution of the mosquitocidal Cry19Aa toxin by rational design. The Cry19Aa toxin, active against mosquito species such as *An. quadrimaculatus* (LC_50_ 3 ng/mL) and *C. pipiens* (LC_50_ 6 ng/mL), showed low activity against *Ae. aegypti* (LC_50_ 1.4x10^5^ ng/mL). After in silico modeling of the Cry4Ba structure and Cry19Aa using Cry3Aa and Cry4Aa as templates, the following changes in Cry19Aa toxin were introduced: (i) Cry19Aa Loop 1 (^355^SYWT^358^) was substituted by the Cry4Ba loop 1 (^332^YQDLR^336^), and (ii) Cry19Aa loop 2 (^414^YPWGD^418^) was completely deleted to mimic the length present in the Cry4Ba toxin. The resulting mutant 19AL1L2 was >42,000 times more toxic to *Ae. aegypti* (LC_50_ 3.3 ng/mL) than the parental toxin Cry19Aa, being one of the highest activity enhancements in the history of 3D-Cry toxin evolution. The rationale behind these substitutions was to test the hypothesis that changing loop sequences involved in receptor binding could be useful to enhance toxicity by increasing the affinity of the toxin to its receptor. Unfortunately, no differences between Cry19Aa and 19AL1L2 were detected in either reversible or irreversible binding to BBMVs, so the hypothesis was not correct, but it was useful to demonstrate that “in vitro evolution” of Cry-toxins could be efficiently performed by rational design.

However, the greatest achievement in 3D-Cry toxin evolution by rational design was that reported by Liu and Dean in 2006 [105]. These authors were able to redirect the toxicity of a lepidopteran active toxin toward an insect from a different order, the dipteran *C. pipiens*. The mutant was constructed using the lepidopteran active Cry1Aa toxin as a scaffold, and changing loops 1 and 2 of the molecule. Loop 1 from Cry1Aa (^311^RG^312^) was enlarged and substituted by the Loop 1 (^332^YQDL^335^) from the mosquito active toxin Cry4Ba (although not active against *C. pipiens*). In addition, part of loop 2 in Cry1Aa (LY^367^RRIILGSGPNNQ^378^) was deleted (LY^365^RRIIL), and the ^376^NNQ^378^ sequence was replaced by a single G, resulting in the mutant 1AaMosq (L1:^311^YQDL^314^; L2:^367^GSGPG^371^), which gained activity against *C. pipiens* while the activity against Lepidoptera *M. sexta* was abolished. The novel toxin 1AaMosq showed an LC_50_ of 45.73 μg/mL when bioassayed against *C. pipiens*, while the parental toxin Cry1Aa was not toxic to this mosquito at toxin concentrations of 100 μg/mL.

A similar rational design performed in the loops of the Domain II has recently been reported in another 3D-Cry toxin [106]. Cry1Ah and Cry1Ai show high sequence similarity (77% identity at amino acid level) but very different specificity. Cry1Ah is toxic to *H. armigera* but non-toxic to *B. mori* and conversely, Cry1Ai is highly toxic to *B. mori* but has no activity against *H. armigera*. As loops in Domain II of the 3D-Cry toxins are involved in specificity, the authors exchanged loops in the two toxins by reverse PCR in order to evolve the activity of Cry1Ai toward *H. armigera*. One of the obtained mutants, Cry1Ai-h-loop2 (Cry1Ai toxin with loop 2 from Cry1Ah toxin), showed a change of specificity. Toxicity against *H. armigera* increased more than 7.8-fold (from LC_50_ > 500 μg/g to 64.23 μg/g). When the exchange was done in loop 2 and loop 3, the resulting Cry1Ai-h-loop2&3 mutant showed an increase in toxicity even higher, around 58 times (from LC_50_ > 500 μg/g to 8.61 μg/g).

Domain I has also been the subject of evolution by rational design [107]. Through bioinformatic analysis, it was found that the first 42 amino acids of Cry2A toxins interacted with a predicted transmembrane (TM) domain (amino acids 51–62) in helix 2 of the toxin. In addition, it was observed that the predicted TM in Cry2A was shorter than the equivalent TMs domains found in other Cry toxins, as a consequence of the presence of two lysines at positions 63 and 64. They predicted that this interaction could be the reason for the Cry2A toxin being less active against lepidopteran pests compared to Cry1A type toxins, so they made the hypothesis that by removing these 42 amino acids from the molecule, a Cry2A variant with increased activity could be obtained. The deleted mutant, D42, showed an increase in activity ranging from 2–3-fold toward Lepidoptera *S. littoralis*, *H. armigera*, and *Agrotis ipsilon*. In addition, when lysines 63 and 64 were substituted by a conserved amino acid present in other toxins (F and P), making the TM domain as long as the one present in other Cry toxins, the activity increased even further. Lysine 63 and 64 in the deleted mutant D42 were replaced by phenylalanine/proline by site-directed mutagenesis, and the mutant toxins D42/K63F, D42/K64F, D42/K63F/K64F, and D42/K63F/K64P were obtained. Single mutant toxins showed the same toxicity as D42, but double mutants increased their toxicity toward the tested Lepidoptera between 1.3 and 2.3 times compared to D42 toxicity, and between 4 and 6.5 times compared to the Cry2A wild type, as predicted.

Another successful example of rational design was performed on the Cry3A toxin [84]. In this work, a chymotrypsin/cathepsin G site (AAPF) was introduced into the loop region between α-helix 3 and α-helix 4 in Cry3A Domain I in order to increase the proteolytic efficiency and hence toxicity. The resulting mutant, mCry3, with a loop sequence ^153^NPAAPFRN^160^, was active against *D. virgifera virgifera* larvae (LC_50_ 65 μg/mL) compared to the residual activity of the parental toxin Cry3A (LC_50_ >> 100 μg/mL). The authors determined that the increase in activity was due to several factors such as a higher solubility at neutral pH, an increase in the efficiency of the proteolytic process, and an increase of specific membrane binding. The introduced mutation did not alter the activity against the Colorado potato beetle larvae, a susceptible insect for Cry3A, but extended activity toward other coleopteran (*D. virgifera virgifera*).

### 2.5. Evolution by Random Mutagenesis

Random mutagenesis is one of the strategies that molecular biologists have available to obtain protein variants. In opposition to site-directed mutagenesis, the position of the mutation in random mutagenesis is not controlled. It is frequently used to discover relevant amino acids in a protein when not enough information on the function of the protein is available [90,108], or to create novel variants of a protein with novel functions. There are several techniques that have been used to perform random mutagenesis in 3D-Cry toxins such as in vitro DNA amplification with degenerated primers and error-prone PCR.

The development of the synthesis of degenerated oligonucleotides in 1988 [109] allowed researchers to introduce random mutations in a specific region of the DNA. In 1999, Kumar et al. [110] used a mixture of degenerated primers for the random mutation of a 3D-Cry toxin using a M13mp19 system that provided ssDNA. The researchers’ objective was to introduce variability at α-helix 4, and at the loop between α-helix 4 and α-helix 5 of the Cry1Ac toxin. For that, a mix of primers with the wild-type sequence (97% of the primers) and degenerated oligonucleotides (3% of the primers) was used to ensure only one mutation per cycle of amplification. Using this technique, the authors obtained the mutant F134L with a 3-fold enhanced toxicity toward *M. sexta* and *H. virescens*.

Error-prone PCR is an in vitro evolution technique that generates variants using the property of the *Taq* polymerase of introducing substitutions in the DNA amplification process when subjected to certain conditions. Error-prone PCR is the most common method for creating combinatorial libraries based on a single gene. Since its development in 1989 [111], it has been used in many applications, not only to mutate DNA codifying for proteins, but also non-coding DNA regions such as promoter regions [112]. The technique is simple and only requires a PCR mixture slightly different from a standard PCR. The gene subjected to mutagenesis is amplified by upstream and downstream primers in a reaction mix containing Mn^2+^ ions, an unbalanced ratio of dNTPs, and/or a higher concentration of Mg^2+^. Depending on the conditions, the overall mutagenesis rate can be controlled. Error-prone PCR mutagenesis has not been extensively used in obtaining Cry toxin variants. Only a few examples of using this technique are found in the bibliography, even though the technique has rendered improved versions of a 3D-Cry toxin. This was the case of the work reported by Shu et al. [113] made in Cry8Ca2 toxin (accession number AY518201), which was active against *Anomala corpulenta*. Two mutants (M100 and M102) showed 5- and 4.4-fold increases in toxicity, respectively, against the larva of this Coleoptera (LC_50_ 0.23 × 10^8^ CFU/g and 0.26 × 10^8^ CFU/g, respectively, compared to LC_50_ 1.15 × 10^8^ CFU/g of the parental toxin). The sequence analysis of the novel variants showed that only a single mutation in each mutant (E642G in M100 and Q439P in M102) was enough to enhance toxicity.

A more recent example of the use of error-prone PCR, although combined with other techniques, is the random evolution of Cry1Ac5, rendering the T525N mutant with a slight increase in toxicity (1.5-fold) toward *S. exigua* [114].

### 2.6. Evolution by Mixing Cry Genes: DNA Shuffling, In Vitro Recombination, and StEP (Staggered Extension Process)

**Figure 2:**
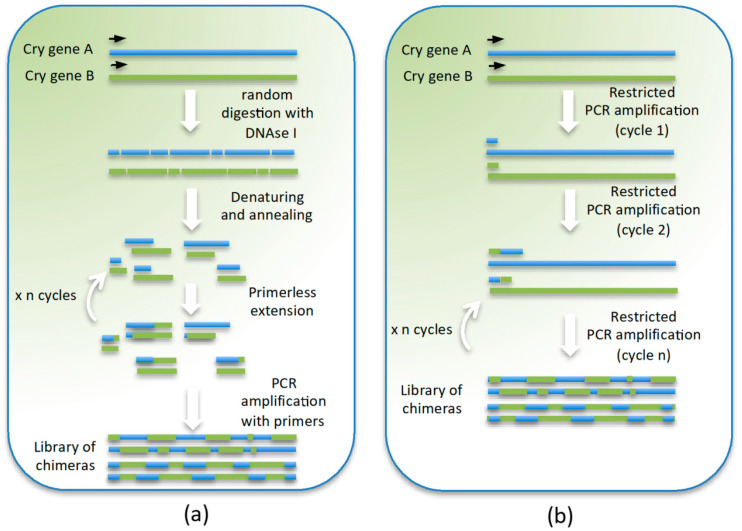
In vitro recombination techniques. (**a**) In DNA shuffling [116], two or more homologous genes are randomly digested with DNAse I (only one strand is represented for simplification purposes). The resulting fragments are extended in a primer-less PCR using homologue fragments as templates. Finally, a PCR using flanking primers is performed in order to obtain a full size library of chimeras, after few rounds of primer-less extension. (**b**) In the staggered extension process or StEP [120], two homologous genes are PCR amplified under restricted conditions (short extension times, and low extension temperature). In cycle 1, a short fragment is extended from a primer in both genes (only one strand is represented). After denaturing, the generated fragments can anneal in the opposite homologous gene, and be extended in cycle 2. After cycle n, a library of recombined chimeras is generated.

DNA shuffling is a powerful in vitro evolution tool for generating artificially and highly diversified sequences by homologous gene recombination (Figure 2a). Although this technique is normally used in proteins of unknown three-dimensional structure, it can be used for any protein. The technique involves random DNA fragmentation of two or more homologous genes with DNAse I, and fragment reassembly in a primer-less PCR. After the generation of a variant library, a screening and selection process of functional variants is conducted.

Since the development of this technique [115,116], DNA shuffling has been used to evolve thousands of proteins, mainly enzymes, to modify their function or activity. The convenience of this powerful technique has also been applied for the in vitro evolution of several 3D-Cry toxins.

Although in vitro evolution by DNA shuffling has not been always successful [117], and sometimes no improved 3D-Cry toxins have been obtained, in several other cases, it has been a suitable technique for obtaining variants with higher activity. For example, DNA shuffling of *cry11Aa*, *cry11Ba*, and *cry11Bb* genes, codifying for toxins active against *Ae. aegypti* and *C. quinquefasciatus*, rendered a mutant (Variant 8) 3.8 times more toxic toward *Ae. aegypti* than the parental toxin Cry11Bb (LC_50_ 22.9 ng/mL), and 6.09 times more toxic than the Cry11Aa toxin (LC_50_ 36.9 ng/mL) [118]. DNA sequence analysis of Variant 8 showed that the mutant contained a deletion of 219 nucleotides (73 aa) at the N-terminal end of the molecule (Domain I), and 6 and 13 nucleotide substitutions in Domain II and III, respectively. The comparative analysis at the protein level between Variant 8 and its parental toxins (Cry11Aa and Cry11Bb) showed 13 amino acid substitutions (GenBank access number MH068787).

Very recently, DNA shuffling has been used to increase the toxicity of an already improved mutant derived from Cry3Aa toxin [52]. The mutant IP3-1, engineered by rational design, contained 15 mutations over the three domains of the toxin (W106L, M117I, V140F, I186V, F206L, K230H, S258T, P292S, E294G, F346L, G468A, L491F, M503T, R531G, and I593M) and showed a higher activity toward *D. virgifera* (LC_50_ 214 ppm) than the parental toxin. To further increase its activity, in vitro evolution by DNA shuffling was carried out and six novel mutant toxins (IP3-2, IP3-3, IP3-4, IP3-5, IP3-6, IP3-7) showed more activity than the parental toxin IP3-1 (LC_50_ 19, 14.7, 13.7, 11.3, 11.6, 7.3 ppm, respectively). An analysis of their sequences showed that mutant toxins contained between six and nine additional mutations. From the six mutants obtained, the IP3-7 variant showed the best increase in toxicity of all (LC_50_ from 214 to 7.33 ppm). Most of the mutations obtained after DNA shuffling resulted in a reduction of positively charged residues such as lysine and arginine, making novel toxins more acidic and more soluble at neutral pH (*D. virgifera* gut juice is weakly acidic) and hence more active. In addition, all the selected variants showed a similar mutation at two different positions (K152E and R158E), located in the α-helices 3 and 4 loop. According to the authors, these mutations made the loops more resistant to *D. virgifera* gut proteases, contributing to the increase in toxicity compared to the parental toxin IP3-1.

A more sophisticated way of obtaining chimeras is by in vitro recombination using the technique called in vitro template-change PCR. With this strategy, a library of recombinant toxins made from the lepidopteran active toxin Cry2Aa (active toward *Ostrinia furnacalis*, *P. xylostella*, *Chilo suppressalis*, and *H. armigera*) and the low toxicity Cry2Ac [119] was made. The strategy involved four steps: (i) ssDNA amplification of the complementary strand of both genes by asymmetric PCR (amplification using a single reverse primer); (ii) synthesis of the coding strand using the ssDNA from gene 1 as a template in the presence of ddATP, which avoids further extension once it is incorporated in the polymerized strand; (iii) DNA extension of the randomly truncated library using gene 2 as a template, which is achieved thanks to the use of the KOD DNA polymerase, shows 3′–5′ exonuclease proofreading activity, and is able to remove the ddATP from the truncated molecule and carry on with the extension of the DNA strand; and (iv) amplification of the full toxin fragment with flanking primers, cloning, and expression in a heterologous system. With this strategy, the authors obtained 37 chimeras (named R1–R37) showing recombination events at 37 different regions of the toxin. When recombination occurred at Domain I or Domain III, no change in specificity was observed. However, when recombination took place at Domain II, toxin specificity drastically changed. The Cry2Ad toxin gained toxicity toward *O. furnacalis* when recombination was in the ^416^NY^417^ region (recombinant R24), toward *P. xylostella* when it occurred at ^440^RPL^442^ (recombinant R26), and toward *C. suppressalis* and *H. armigera* when it took place at ^455^GTPGGA^460^ (recombinant R27).

The technique, called the staggered extension process or StEP [120], has the same objective as DNA shuffling of producing an in vitro recombination of two or more genes, but with a slightly different methodology. In this technique (Figure 2b), two (or more) homologous genes are denatured and extended from the same primer, using a thermo-cycling program in which the extension step is highly limited in time (few seconds (5 s)) and temperature (extension is carried out at 55 °C, temperature in which *Taq* polymerase has a low extension rate). After 70–80 cycles of denaturing and priming-extension, a library with full-length recombined genes that is cloned and screened can be obtained. This technique has been used for in vitro evolution of the active part (DI, DII, and DIII) of the Cry1Ac5 toxin (GenBank acc. Number M73248) in combination with error-prone PCR. The variants were cloned into a plasmid containing the pro-toxin C-terminal end by Red/ET homologous recombination [121,122]. From the 57 variants obtained, only one was expressed as a full-length 130 kDa toxin containing the mutation T524N in the β-16 and β-17 loops in Domain III. The variant produced more crystals than the wild type, but slightly smaller. When bioassayed toward *S. exigua*, a toxicity 1.5 times higher (LC_50_ 9.6 μg/mL compared to 14.1 μg/mL of the wild type) was observed.

### 2.7. Evolution by Phage Display

**Figure 3:**
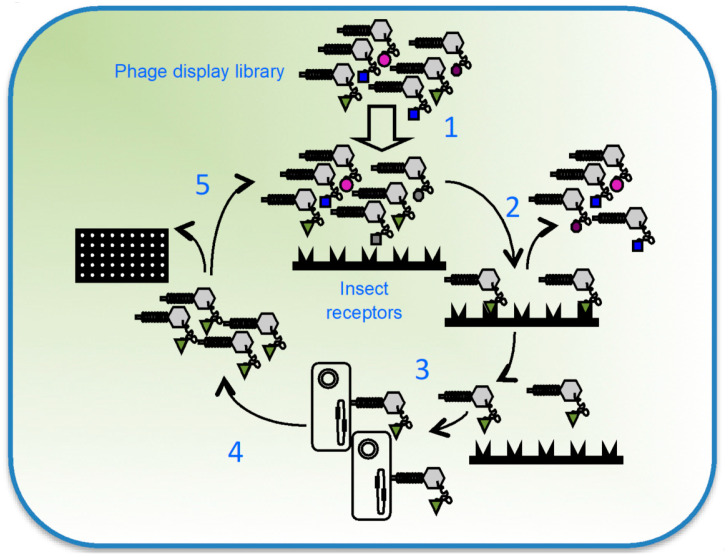
Biopanning of a phage display toxin library. The phage displayed toxin library is biopanned against a specific insect receptor (1). Those phage displaying toxins with affinity to the insect receptor will be retained and those without affinity will be washed out (2). Bond phage will be recovered (3) and amplified (4) by a susceptible *E. coli* strain, making possible to repeat the process (5) in order to obtain higher affinity toxins from the selected pool.

As has been reviewed thus far, generation of 3D-Cry toxin variants does not represent a big challenge nowadays as many molecular techniques for constructing libraries with a high number of mutants are available. The real challenge is to find which of the variants in the library is useful and has the desired properties. Therefore, the key question of in vitro evolution of a protein is the library screening. In the particular case of 3D-Cry toxins, this screening is labor-intensive as every single mutant has to be expressed, solubilized, and bioassayed, representing a time consuming task. As a consequence, only a reduced number of variants from the library are normally tested, and on many occasions, no improved mutants are found. To overcome this problem, approaches such as phage display have been explored to provide a means for the selection of potentially useful 3D-Cry mutants. Phage display is a molecular tool for the screening of library variants with a specific binding characteristic. A phage displayed protein (or a mutant library) consists of its expression on the surface of a bacteriophage in such a way that the protein is available to interact with other proteins, while it is bound to the virus. Display is achieved thanks to the fusion of the protein of interest (or library) to one of the proteins on the surface of the phage. When the phage replicates and viral particles assemble, the protein of interest is also assembled on the surface, being available to interact with other proteins. Displayed libraries can be screened by a process called biopanning, a methodology that allows for the selection of those phages with the desired binding properties (Figure 3). As the phenotype of the selected variant and the genotype in a phage display system are linked, once a phage is selected, the coding DNA for the protein variant can be obtained from the phage genome. Given that one of the premises for 3D-Cry toxin toxicity is to bind to a receptor, this makes phage display a suitable molecular tool for the screening of variants with novel binding characteristics.

Since the invention of the phage display technique [123] and its use for the selection of a peptide from a library with an antibody, many other applications have been developed [124,125,126,127]. 3D-Cry toxins have also benefited from the advantages of phage display technology, although several technical limitations had to be overcome before the methodology could be used for big proteins such as 3D-Cry toxins. The first attempt at displaying a 3D-Cry toxin on the surface of a phage was reported by Marzari et al. [128] using the Cry1Aa toxin and M13 phage. Unfortunately, the toxin was not properly displayed and deletions on the fusion protein were observed. One year later, Kasman et al. [129] reported the successful display of the Cry1Ac toxin on the surface of the M13 phage, although the toxin was unable to bind to the APN receptor in in vitro experiments. Later on, other display systems based on the λ and T7 phage, which are assembled in the cytoplasm of *E. coli* and released after lysis instead of being secreted through the bacterial membrane as in M13, were proven to be more appropriate for 3D-Cry toxins. The first successful phage display system, in which the 3D-Cry toxin was able to bind to natural receptors, was described by Vilchez et al. [130]. In this study, the Cry1Ac1 toxin was fused to the gpD protein, an auxiliary protein that represents one of the major components of the λ phage capsid. The displayed toxin was able to selectively recognize and bind proteins present in *M. sexta* BBMVs. Later on, other display systems using the T7 phage [131] and M13 [132] were described. Once the problem of displaying a 3D-Cry toxin was overcome, library variants were developed.

Mutant libraries have been constructed in specific areas of the 3D-Cry toxin using several molecular approaches such as degenerated primers [133,134,135], DNA shuffling [136,137], or using a previously constructed antibodies library [138]. All these libraries were screened for variants showing high binding affinity toward two of the most well-known receptors (cadherin like receptor and APN), and although an increase of binding affinity is not a guarantee for increased toxicity [134,135], some authors have managed to obtain enhanced 3D-Cry toxin variants compared to the parental toxin.

The first successful report describing the use of a phage display library for the evolution of a 3D-Cry toxin was made by Ishikawa et al. [133] using the T7 phage. They constructed a library of Cry1Aa1 variants at the loop 2 of Domain II, one of the main determinants for specificity in 3D-Cry toxins. Loop 2 variants were constructed by PCR with the degenerated primer Aa369(IILGSGP)375-degenerate-sense (5′TTATATAGAAGANNNNNNNNNNNNNNNNNNNNNAATAATCAGGAACTGTTTG3′), which could theoretically introduce 1.28 × 10^9^ possible combinations on the seven amino acid residues of the loop. However, in practice, the library contained only 5.0 × 10^5^ variants, less than 0.04% of the possible mutations. Despite the reduced number of variants, the authors managed to select a toxin mutant (R5–51) with strong binding affinity to the bead-immobilized cadherin-like protein BtR175. The selected variant, R5–51, was four times more toxic (LC_50_ 1.6 μg/g diet) than the Cry1Aa1 wild type toxin (LC_50_ 6.3 μg/g diet) toward *B. mori*.

Another case of success in the quest of improving the toxicity of a natural 3D-Cry toxin by phage display technology was the in vitro evolution performed on the moderately active Cry8Ka1 toxin toward the Coleoptera *Anthonomus grandis* [137]. This time, variability was obtained by *cry8Ka1* gene shuffling, which was cloned in the pComb3X phagemid [139] fused to the pIII protein in a M13 system. The resulting library, pCOMBcry8Ka1var, containing 1.0 × 10^5^ cfu/mL variants, was screened toward *A. grandis* BBMVs. Biopanning rendered one variant (Cry8Ka5 mutant) that showed a 3-fold increase in toxicity. Sequence analysis of the Cry8Ka5 variant demonstrated that mutations were randomly introduced at different positions in Domain I (R82Q), Domain II (Y260C, P321A), and Domain III (R508G, K538E, E594N) of the toxin. In addition, a deletion of 16 residues at the N-terminal end was observed.

Non-natural 3D-Cry toxins have also been evolved by DNA shuffling and phage display technology [136]. This is the case of the Cry1Ia12synth toxin (NCBI gene bank accession number FJ938022), a synthetically derived toxin from Cry1Ia12 with a modified codon usage for plant expression optimization. Cyr1Ia12synth is toxic for *S. frugiperda*, but not for the sugarcane giant borer *Telchin licus licus*. From the 30 variants selected by phage display using *T. l. licus* BBMVs, four of them showed higher activity toward *T. l. licus* compared to the wild type toxin. Variant 1 (D233N, E639G), variant 2 (D233N), variant 3 (I116T, L266F, K580R), and variant 4 (M45V, D233N) showed 61%, 75% 56%, and 58% mortality, respectively, higher than the wild type and the negative control (25% mortality). This represents an example of the in vitro evolution of a 3D-Cry toxin in order to be active toward another Lepidoptera specie.

The latest report of a 3D-Cry toxin in vitro evolution using phage display technology was by Dominguez-Flores et al. [140]. In this case, a library of “Crybodies” was displayed on a λ phage system similar to the one reported by Vilchez et al. [130]. Crybodies are molecules derived from the lepidopteran active Cry1Aa13 toxin where loop 2 of Domain II has been replaced by the hypervariable region contained at the complementary determinant region 3 (CDR-H3) of a human antibody library [138]. The Crybody library was biopanned to *Ae. aegypti* larvae guts homogenates, and the selected phage, with high affinity toward gut proteins, was used to obtain the novel Crybodies. Crybodies Cry1Aa13-A8 (L2:^367^GAREGSSSAYDYW^379^) and Cry1Aa13-A12, (L2:^367^GARGDPDFDHSTSYYLDYC^385^) showed significant mortality (around 90%) after 120 h at 20 μg/mL, while no toxicity was observed in the parental Cry1Aa13 toxin. Concomitantly, both variants showed a 50% decrease in toxicity toward their natural lepidopteran target (*B. mori*). In this case, phage display was proven to be useful not only for improving toxicity against an insect or related species, but also to select variants active against insects from a different order.

Another example that used phage display technology in the field of 3D-Cry toxin evolution is the work reported by Shao et al. [141]. This work describes the construction of six 3D-toxin mutants, obtained by replacing loop 1, loop 2, and loop 3 in Domain II of the Cry1Ab toxin with what they called “gut-binding peptides” or GBPs. These peptides were obtained from a random peptide library displayed on a phage that was biopanned against BBMVs obtained from the hemipteran *Nilaparvata lugens* [141]. P2S (CLMSSQAAC) and P1Z (CHLMSSQAAC) were introduced by overlapping PCR in substitution of loop 1 (^278^RG^279^), loop 2 (^335^RRPFNIGINNQ^345^), and loop 3 (^401^SMFRSGFSNSSVS^413^) in the Cry1Ab toxin. *N. lugens* nymph bioassays showed increased toxicity in five of the six variants selected. Only mutant L3-P1Z was less toxic than the wild type (with an LC_50_ of 189.83 μg/mL). The rest of the mutant toxins (L1-P2S, L2-P2S, L3-P2S, L1-P1Z, and L2-P17) were 5, 9, 5, 1.4, and 2.5 times more toxic, respectively, than the parental toxin. Substitution of loops 1, 2, and 3 was concomitant with a loss in toxicity of Cry1Ab toward *P. xylostella*. This work demonstrated that the in vitro evolution of Cry toxins is not only restricted to the selection of variants with an improved binding to natural receptors, but also evolution can be directed to bind other molecules in the insect guts.

### 2.8. Evolution by PACE (Phage-Assisted Continuous Evolution)

**Figure 4:**
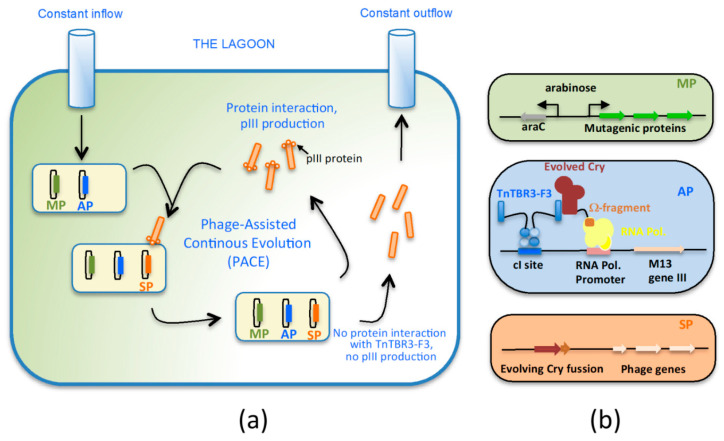
Phage assisted continuous evolution (PACE). In the PACE technique, the evolution occurs in a continuous culture of a highly engineered *E. coli* strain called the lagoon (**a**). The *E. coli* strain contains three plasmids (**b**), the mutagenic plasmid (MP), containing mutagenic proteins induced by arabinose, the selection plasmid (SP), which contains all the M13 genes for phage replication, except for *gene III*, and a transcriptional fusion of the evolving *cry* gene toxin and the *rpoZ* gene, codifying for the omega sub-unit of the RNA polymerase, and the accessory plasmid (AP). AP plasmid contains the M13 *gene III* downstream of a cI site and a promoter. The fusion protein between cI and a fragment from *T. ni* cadherin like receptor (TnTBR3-F3) binds to the cI site. Only Cry toxin variants interacting with TnTBR3-F3 will bind to the proximity of the promoter site so the *gen III* expression will be possible, rendering viable infecting M13 particles. However, if Cry toxins with no affinity toward the TnTBR3-F3 fragment are produced, *gene III* will not be expressed and no infecting M13 particles will be generated. Reproduced from [146] Copyright 2016, Springer Nature.

Phage-assisted continuous evolution is a technique developed at Harvard University by David L. Liu´s research group [142]. It is one of the latest techniques developed in the field of the in vitro evolution of proteins. It is a complex technique, that, as its name implies, is performed with the assistance of a phage. Strictly speaking, it is not an in vitro technique, as evolution is performed inside of a highly engineered *E. coli* strain, but as it is performed in a laboratory, it is considered to be an in vitro evolution technique. The evolution is carried out in what is called “the lagoon” (Figure 4a), an *E. coli* culture with a constant inflow and outflow of growing media. The flow is set up at the appropriate speed to serve as the selection process for the mutants generated in the lagoon, as only the suitable and fast growing mutants stay in the lagoon, while the non-useful mutants are washed out from the growing flask. The average residence time of the cells is less than the *E. coli* replication time. The *E. coli* strain where the evolution takes place contains three plasmids (Figure 4b): (i) an arabinose-inducible mutagenic plasmid (MP), that contains proteins that disrupt the proofreading activity of DNA polymerases, so increasing the error rate in replication; (ii) a selection plasmid (SP), that contains the protein to be evolved and all the genes necessary for M13 phage replication except for *gen III*, essential for host infection; and (iii) an accessory plasmid (AP) where the essential gen III for M13 phage replication is expressed, but only if the right mutant is generated. With this system, the mutation process and the selection process are coupled as only the desired mutants allowing the expression of protein III are replicated. Non-useful mutations produce non-infective phage, so they will be unable to reproduce and will be washed out from the lagoon. The system solves the cumbersome need to screen the entire library in each round of evolution and, given the life cycle of M13 is just 10 min, a high number of rounds of protein evolution could be conducted in a single week. PACE has been used to evolve proteins such as polymerases [143], proteases [144], and genome-editing proteins [145], obtaining variants with completely novel activities and specificities.

PACE has recently been adapted for the evolution of a 3D-Cry toxin, specifically the lepidopteran Cry1Ac toxin [146]. The cabbage looper *T. ni*, naturally susceptible to Cry1Ac, has developed resistance to the toxin, a fact that has been associated with the mutation of the *ABCC2* transporter gene [147] and the downregulation of the expression of *APN1* [148]. There is no evidence that *T. ni* uses any cadherin-like receptors for its function, so the objective of this work was to evolve a Cry1Ac toxin to specifically bind to a cadherin-like protein (TnCAD), present in the insect cell membrane of *T. ni*, in order to be used as a toxin receptor. For that, the SP plasmid contained, apart from all phage genes (except for *gen III*), a transcriptional fusion of *cry1Ac* with the *rpo*Z gene codifying for the omega sub-unit of the RNA polymerase (Figure 4b). The omega sub-unit is essential for the activity of the RNA polymerase and unless it is present in the RNA polymerase enzymatic complex, the transcription is not possible. The AP plasmid contains M13 *gen III* with an upstream promoter region for the RNA polymerase, and the binding site for the cI protein, a phage repressor protein. In addition, a transcriptional fusion between the cI protein and a fragment of the TnCAD cadherin-like protein, called TnTBR3-F3, was included in the AP plasmid. This fusion protein is able to recognize the cI binding site, allowing the TnTBR3-F3 receptor to interact and bind to other proteins.

In this system, if a Cry toxin variant has the ability of interacting with the TnTBR3-F3 receptor as a consequence of the generated mutations, then it will bind at the promoter region of the M13 *gen III* through the omega sub-unit of the RNA polymerase, allowing the expression of the essential *gen III* for phage replication. If the introduced mutation is not suitable for TnTBR3-F3 binding, then the mutant will not be present in the promoter region, the protein III will not be produced, and the resulting phage will not be infective and it will be lost. The PACE system adapted to the evolution of a Cry1Ac toxin, rendered A01s, C03s, and C05s variants with high binding affinities to the membrane protein TnCAD, in opposition to the wild type toxin Cry1Ac. In addition, when these toxins were bioassayed against *T. ni*, they were 2.2, 1.1, and 1.8 times more active compared to the wild type Cry1Ac, respectively, indicating that toxin-receptor evolution had been taking place. Furthermore, when A01s, C03s and C05s were bioassayed against Cry1Ac resistant *T. ni*, toxin variants showed an increase in toxicity of 334-, 27.8-, and 26.4-fold compared to the wild type Cry1Ac toxin. This work represents a proof of concept that evolution of 3D-Cry toxins to bind novel receptors is possible through PACE and that the technique could be useful in cases where insects have developed resistance to natural toxins.

## 3. Concluding Remarks

The use of several molecular techniques has allowed researchers to obtain 3D-Cry toxin mutants with improved activities compared to natural toxins. Although in many cases the reason behind this enhancement is not known, the reality is that molecular techniques have been proven to be useful to develop artificial variants. From a practical point of view, these variants represent a real alternative to (i) the intrinsic limitation that 3D-Cry toxins show, as they are only active against a narrow range of insects, and (ii) the resistance phenomena that insects have experienced as a consequence of the extensive use of natural 3D-Cry toxins. This report is proof that minimal changes in the amino acid sequence of a 3D-Cry toxin can lead to a great improvement in toxicity, and that protein engineering, rational design, and in vitro evolution are powerful tools to develop artificial 3D-Cry toxins with surprising and novel activities. The compilation of all of these successful examples and the description of all the sensitive positions that have been used to obtain 3D-Cry toxin variants represents a valuable source of information for the further manipulation of natural toxins.

## Figures and Tables

**Table 1:** Compilation of all the improved toxin mutants obtained throughout the molecular evolution of 3D-Cry toxin history. Parental toxin, mutants, and results obtained, together with the methodology used for protein evolution, are detailed in chronological order.

Molecular Technique Used	Parental Toxin Evolved	Mutant Name	Evolution Result (Insect)/Evolution Level ^1^	Activity Enhancement	Domain Evolved ^2^	Reference
Random mutagenesis with mutagens	CryIA(b)	P26-3 P48a14 P48c5 P36a65 P95a76 P95a86 P98c1 P99c62 P107c22 107c25 114a30	Enhanced toxicity (*H. virescens*)/SS	3–5-fold	DI *	[68]
Homolog-scanning mutagenesis	ICPC73	OSU 4205	Novel activity (*B. mori*)/SL		DII *	[70]
Homolog-scanning mutagenesis	CryIIB	Hybrid 513	Novel activity (from Lepidoptera to a dual Lepidoptera and Diptera)/OL		DII *	[73]
Domain swapping (in vivo recombination)	CryIA(a) and CryIA(c)	pHy32 pHy45 pHy104	Enhanced toxicity (*T. ni* and *Heliothis* sp)/SS Novel activity (*S. littoralis*)/SL	2–37-fold 3–7-fold	DII *	[76]
Homolog-scanning mutagenesis	CryIA(c)	Hybrid 4109	Enhanced toxicity (*H. virescens*)/SS	30-fold	DII *	[74]
Site directed mutagenesis	CryIA(c)	H168R	Enhanced toxicity (*M. sexta*)/SS	3–5-fold	DI	[87]
Rational design (site directed mutagenesis)	Cry4B	R203A	Enhanced toxicity (*Ae. aepypti*)/SS	2.8-fold	DI	[102]
Domain swapping (in vivo recombination)	CryIE	G27	Enhanced toxicity (*S. exigua*)/SS	>50-fold	DIII	[77]
Domain swapping (cloning)	CryIA(b)	H04	Enhanced toxicity (*S. exigua*)/SS	More than 60-fold	DIII	[78]
Site directed mutagenesis (alanine scanning mutagenesis)	CryIIIA	Triple mutant: S484A, R485A, G486A	Enhanced toxicity (*T. molitor*)/SS	2.4-fold	DII (Loop 3)	[91]
Site directed mutagenesis	Cry1Ab	N372A N372G	Enhanced toxicity (*L. dispar*)/SS	8.53-fold 9.61-fold	DII	[92]
Site directed mutagenesis	Cry1Ab	DF-1: Triple mutant N372A, A282G, L283S	Enhanced toxicity (*L. dispar*)/SS	36-fold	DII	[92]
Domain Swapping (cloning)	Cry1C	Cry1C/Ab hybrid	Enhanced toxicity (*S. littoralis*, *O. nubilalis,* and *P. xylostella*)/SS	3-, 4- and 35-fold respectively	DI-DVII	[81]
Random mutagenesis	Cry1Ac1	F134L	Enhanced toxicity (*M. sexta* and *H. virescens*)/SS	3-fold	DI α-Helix 4)	[110]
Domain swapping (in vivo recombination)	Cry1Ba	BBC13 BBC15	Enhanced toxicity (*M. sexta*)/SS Enhanced toxicity (*S. exigua*) /SS Enhanced toxicity (*M. sexta*) /SS	11.8-fold 8.3-fold 7.8-fold	DIII	[79]
Domain swapping (in vivo recombination)	Cry1Fa	FFC1	Enhanced toxicity (*S. exigua*) /SS	5.5-fold	DIII	[79]
Rational design (site directed mutagenesis)	Cry3A loop 1	A1	Enhanced toxicity (*T. molitor*) /SS	11.4-fold	DII	[93]
Rational design (site directed mutagenesis)	Cry3A loop 1	A2	Enhanced toxicity (*T. molitor*)/SS	2.7-fold	DII	[93]
Domain swapping (cloning)	Cry1Ia	1Ia/1Ia/1Ba hybrid	Enhanced toxicity (*L. decemlineata*)/SS	2.5-fold respect Cry1Ia and 7.5-fold respect Cry1Ba	DI, DII, DIII	[82]
	Cry1Ba	1Ba/1Ia/1Ba hybrid	Enhanced toxicity (*L. decemlineata*)/SS	17.9-fold	DI, DII, DIII	[82]
Rational design (Site directed mutagenesis)	Cry4Ba using Loop3 from Cry4Aa	4BL3PAT	Evolution from *Anopheles* and *Aedes* to *Culex*/SL	700- and 285-fold increase	DII	[103]
Rational design (Site directed mutagenesis)	Cry19Aa using loop from Cry4Ba	19AL1L2	Evolution from *Anopheles* and *Culex* to *Aedes*/SL	42,000-fold increase	DII	[104]
Domain swapping (in vivo recombination)	Cry1Ca and Cry1Fb using DIII of Cry1Ac	RK15 RK12	Enhanced toxicity (*H. virescens*)/SS Enhanced toxicity (*H. virescens*)/SS	172-fold 69.6-fold		[80]
Rational design (Site directed mutagenesis)	Cry1Aa using loop 1 from Cry4Ba	1AaMosq	Evolution from Lepidoptera to Diptera (mosquito)/OL	From no activity at 100 ug/mL to an LC_50_ 45.73 of ug/mL	DII	[105]
Site directed mutagenesis	Cry1Ab	W73F W210F W219F W455F	Enhanced toxicity (*M. sexta*)/SS Enhanced toxicity (*M. sexta*)/SS Enhanced toxicity (*M. sexta*)/SS Enhanced toxicity (*M. sexta*)/SS	3.3-fold 1.5-fold 2.3-fold 1.4-fold	DI and DII	[88]
Error prone PCR	Cry8Ca2	M100 M102	Enhanced toxicity (*A. corpulenta*)/SS	5-fold 4.4-fold	DIII DII	[113]
Rational design (Site-directed mutagenesis)	Cry2A	D42	Enhanced toxicity (*S. littoralis*)/SS Enhanced toxicity (*H. armigera*)/SS Enhanced toxicity (*A. ipsilon*)/SS	2.85-fold 1.99-fold 2.87-fold	DI	[107]
Rational design (Site-directed mutagenesis)	Cry2A	D42/K63F/K64F	Enhanced toxicity (*S. littoralis*)/SS Enhanced toxicity (*H. armigera*)/SS Enhanced toxicity (*A. ipsilon*)/SS	4.5-fold 2.9-fold 3.7-fold	DI	[107]
Rational design (Site-directed mutagenesis)	Cry2A	D42/K63F/K64P	Enhanced toxicity (*S. littoralis*)/SS Enhanced toxicity (*H. armigera*)/SS Enhanced toxicity (*A. ipsilon*)/SS	6.6-fold 4.1-fold 4.9-fold	DI	[107]
Phage display	Cry1Aa1	R5-51	Enhanced toxicity (*B. mori*)/SS	4-fold	DII (loop 2)	[133]
Site directed mutagenesis	Cry3A	mCry3A	Novel activity (*D. virgifera virgifera*)/SL	From LC_50_ >> 100 μg/mL to 65 μg/ml	DI (Loop α-helix 3 and 4)	[84]
Site directed mutagenesis	Cry1Ac	N546A	Enhanced toxicity (*H. armigera*)/SS	1.8-fold	DIII	[99]
Site directed mutagenesis	Cry1Ab	V171C L157C	Enhanced toxicity (*L. dispar*)/SS	25-fold 4-fold	DI	[89]
DNA Shuffling and Phage display	Cry1Ia12synth	Variant 1 Variant 2 Variant 3 Variant 4	Novel toxicity (*T. licus licus*)/SL	LC_50_ not determined	DI, DII, DIII	[136]
Domain swapping (Overlapping PCR)	mCry3A	eCry3.1Ab	Enhanced toxicity (*D. virgifera virgifera*)/SS	From low toxicity to 93% mortality at 7.5 μg/mL	DIII	[83]
Error prone PCR and StEP shuffling	Cry1Ac5	T524N	Enhanced toxicity (*S. exigua*)/SS	1.5-fold	DIII	[114]
DNA shuffling and phage display	Cry8Ka1	Cry8Ka5	Enhanced toxicity (*A. grandis*)/SS	3-fold	DI, DII, DIII	[137]
Site directed mutagenesis	Cry1Ac5	S581A I585A	Enhanced toxicity (*H. armigera*)/SS Enhanced toxicity (*H. armigera*)/SS	1.72-fold 1.89-fold	DIII	[45]
Rational design (Site directed mutagenesis)	Cry2Ab	N309S F311I A334S	Enhanced toxicity (*An. gambiae*)/SS Enhanced toxicity (*An. gambiae*)/SS Enhanced toxicity (*An. gambiae*)/SS	1.17-fold 3.17-fold 6.75-fold	DII	[94]
Site directed mutagenesis	Cry5Ba	N586A	Enhanced toxicity (*C. elegans*)/SS	9-fold	DIII	[101]
In vitro template-change PCR (TC-PCR)	Cry2Ad	R24 R26 R27 R27	Novel activity (*O. furnacalis*)/SL Novel activity (*P. xylostella*)/SL Novel activity (*C. suppressalis*)/SL Novel activity (*H. armigera*)/SL	From 0% to 26.7% mortality From 4.6 to 75.6% From 6.7 to 76.7% From 2.2 to 84.1%	DII	[119]
Phage display	Cry1Ab	L1-P2S L2-P2S L3-P2S L1-P1Z L2-P1Z	Enhanced toxicity (*N. lugens*)/SS Enhanced toxicity (*N. lugens*)/SS Enhanced toxicity (*N. lugens*)/SS Enhanced toxicity (*N. lugens*)/SS Enhanced toxicity (*N. lugens*)/SS	5-fold 8.9-fold 5-fold 1.4-fold 2.5-fold	DII	[141]
PACE	Cry1Ac	A01s C04s C05s	Enhanced toxicity (*T. ni* /Cry1Ac resistant *T. ni*)/SS Enhanced toxicity (*T. ni* /Cry1Ac resistant *T. ni*)/SS Enhanced toxicity (*T. ni* /Cry1Ac resistant *T. ni*)/SS	2.2/334-fold 1.1/27.8-fold 1.8/26.4-fold	Not available	[146]
Rational design (reverse PCR)	Cry1Ai	Cry1Ai-h-loop2 Cry1Ai-h-loop2&3	Activity redirected from *B. mori* to *H. armigera*/SL	>7.8-fold >58-fold	DII	[106]
Domain swapping	Cry9Aa	Cry1Ac-Cry9Aa Cry1Ac-Cry9AaMod	Enhanced toxicity (*H. armigera*)/SS Enhanced toxicity (*H. armigera*)/SS	4.9-fold 5.1-fold	DI, DII, DIII	[85]
Gene fusion	Chimeric protein Cry4Ba and Cry1Ac	Cry(4Ba-1Ac)	Enhanced toxicity (*Culex pipiens*)/SS	>238-fold	DI-DVII	[21]
Phage display	Cry1Aa13	Cry1Aa13-A8 Cry1Aa13-A12	Activity redirected from *B. mori* to *Ae. aegypti*/OL	From 0% activity to 90% activity at 20 μg/mL	DII	[140]
Site directed mutagenesis (Alanine scanning)	Cry1Ab	S509A V513A N514A	Enhanced toxicity (*S. frugiperda*)/SS	9.5-fold 12.7-fold 51-fold	DIII (β-16)	[97]
Site directed mutagenesis (saturation mutagenesis)	Cry1Ab	N514F N514H N514K N514L N514Q N514S N514V	Enhanced toxicity (*S. frugiperda*)/SS	44-fold 16-fold 7-fold 9-fold 26-fold 23-fold 9-fold	DIII (β-16)	[97]
Site directed mutagenesis (Alanine scanning)	Cry1Fa	N504A	Enhanced toxicity (*S. frugiperda*)/SS	11-fold	DIII (β-16)	[97]
Site directed mutagenesis	Cry1Ca	V509A N510A	Enhanced toxicity (*S. frugiperda*)/SS Enhanced toxicity (*S. frugiperda*)/SS	1.6-fold 1.5-fold	DIII (β-16) DIII (β-16)	[98]
DNA shuffling	Cry11Aa, Cry11Ba, and Cry11Bb	Variant 8	Enhanced toxicity (*Ae. aegypti*)/SS	3.8-fold increase compared to Cry11Bb and 6.09-fold increase compared to Cry11Aa	DI, DII, DIII	[118]
Rational design and DNA Shuffling	IP3-1: an artificial mutant derived from Cry3Aa1	IP3-2 IP3-3 IP3-4 IP3-5 IP3-6 IP3-7	Enhanced toxicity (*D. virgifera virgifera*)/SS	11-fold 14.6-fold 15.6-fold 19-fold 18.4-fold 29.3-fold	DI, DII, DIII	[52]

^1^ Evolution level: SS: Same Specie (evolution toward the same specie; activity enhancement); SL: Specie Level (toxicity evolved to other specie insect from the same order); OL: Order Level (toxicity evolved toward other insect from a different order). ^2^ DI: Domain 1; DII: Domain 2; DIII: Domain 3. Di *: Indicated the domain evolved although it was not known at the time.

## Supplementary Materials

The following are available online at https://www.mdpi.com/2072-6651/12/9/600/s1, Table S1: Sequence of some mutant toxins mentioned in the review.

## References

[1] Dammak M., Jaoua S., Tounsi S. (2011). Construction of a *Bacillus thuringiensis* genetically-engineered strain harbouring the secreted Cry1Ia delta-endotoxin in its crystal. Biotechnol. Lett..

[2] Hannay C. (1953). Crystalline inclusions in aerobic sporeforming bacteria. Nature.

[3] Angus A. (1954). Bacterial toxin paralysing silkworm larvae. Nature.

[4] Ishiwata S. (1901). On a type of severe flacherie (sotto disease). Dainihon Sanshi Kaiho.

[5] Bacterial Pesticidal Protein Resource Center. https://www.bpprc.org.

[6] Crickmore N., Berry C., Panneerselvam S., Mishra R., Connor T.R., Bonning B.C. (2020). A structure-based nomenclature for *Bacillus thuringiensis* and other bacteria-derived pesticidal proteins. J. Invertebr. Pathol..

[7] Crickmore N., Zeigler D.R., Feitelson J., Schnepf E., Van Rie J., Lereclus D., Baum J., Dean D.H. (1998). Revision of the nomenclature for the *Bacillus thuringiensis* pesticidal crystal proteins. Microbiol. Mol. Biol. Rev..

[8] Palma L., Muñoz D., Berry C., Murillo J., Caballero P. (2014). *Bacillus thuringiensis* toxins: An overview of their biocidal activity. Toxins.

[9] Jing X., Yuan Y., Wu Y., Wu D., Gong P., Gao M. (2019). Crystal structure of *Bacillus thuringiensis* Cry7Ca1 toxin active against *Locusta migratoria* manilensis. Protein Sci..

[10] Li J., Carroll J., Ellar D. (1991). Crystal structure of insecticidal delta-endotoxin from *Bacillus thuringiensis* at 2.5 Å resolution. Nature.

[11] Grochulski P., Masson L., Borisova S., Pusztai-Carey M., Schwartz J.-L., Brousseau R., Cygler M. (1995). *Bacillus thuringiensis* CrylA(a) Insecticidal toxin: Crystal structure and channel formation. J. Mol. Biol..

[12] Galitsky N., Cody V., Wojtczak A., Ghosh D., Luft J.R., Pangborn W., English L. (2001). Biological crystallography structure of the insecticidal bacterial δ-endotoxin Cry3Bb1 of *Bacillus thuringiensis*. Acta Cryst..

[13] Derbyshire D., Ellar D., Li J. (2001). Crystallization of the *Bacillus thuringiensis* toxin Cry1Ac and its complex with the receptor ligand N-acetyl-D-galactosamine. Acta Crystallogr. D Biol. Crystallogr..

[14] Morse R.J., Yamamoto T., Stroud R.M. (2001). Structure of Cry2Aa suggests an unexpected receptor binding epitope. Structure.

[15] Boonserm P., Davis P., Ellar D.J., Li J. (2005). Crystal Structure of the mosquito-larvicidal toxin Cry4Ba and its biological implications. J. Mol. Biol..

[16] Boonserm P., Mo M., Angsuthanasombat C., Lescar J. (2006). Structure of the functional form of the mosquito larvicidal Cry4Aa toxin from *Bacillus thuringiensis* at a 2.8-angstrom resolution. J. Bacteriol..

[17] Guo S., Ye S., Liu Y., Wei L., Xue J., Wu H., Song F., Zhang J., Wu X., Huang D. (2009). Crystal structure of *Bacillus thuringiensis* Cry8Ea1: An insecticidal toxin toxic to underground pests, the larvae of *Holotrichia parallela*. J. Struct. Biol..

[18] Hui F., Scheib U., Hu Y., Sommer R.J., Aroian R.V., Ghosh P. (2012). Structure and glycolipid binding properties of the nematicidal protein Cry5B. Biochemistry.

[19] Evdokimov A.G., Moshiri F., Sturman E.J., Rydel T.J., Zheng M., Seale J.W., Franklin S. (2014). Structure of the full-length insecticidal protein Cry1Ac reveals intriguing details of toxin packaging into in vivo formed crystals. Protein Sci..

[20] Pardo-López L., Soberón M., Bravo A. (2013). *Bacillus thuringiensis* insecticidal three-domain Cry toxins: Mode of action, insect resistance and consequences for crop protection. FEMS Microbiol. Rev..

[21] Zghal R.Z., Elleuch J., Ben Ali M., Darriet F., Rebaï A., Chandre F., Jaoua S., Tounsi S. (2017). Towards novel Cry toxins with enhanced toxicity/broader: A new chimeric Cry4Ba/Cry1Ac toxin. Appl. Microbiol. Biotechnol..

[22] Melo A.L.D.A., Soccol V.T., Soccol C.R. (2016). *Bacillus thuringiensis*: Mechanism of action, resistance, and new applications: A review. Crit. Rev. Biotechnol..

[23] Lucena W.A., Pelegrini P.B., Martins-de-Sa D., Fonseca F.C.A., Gomes J.E., de Macedo L.L.P., da Silva M.C.M., Sampaio R., Grossi-de-Sa M.F. (2014). Molecular approaches to improve the insecticidal activity of *Bacillus thuringiensis* Cry toxins. Toxins.

[24] Coates B.S. (2016). *Bacillus thuringiensis* toxin resistance mechanisms among Lepidoptera: Progress on genomic approaches to uncover causal mutations in the European corn borer, *Ostrinia nubilalis*. Curr. Opin. Insect Sci..

[25] Peterson B., Bezuidenhout C., Van den Berg J. (2017). An overview of mechanisms of Cry toxin resistance in Lepidopteran insects. J. Econ. Entomol..

[26] Bravo A., Gómez I., Porta H., García-Gómez B.I., Rodriguez-Almazan C., Pardo L., Soberón M. (2013). Evolution of *Bacillus thuringiensis* Cry toxins insecticidal activity. Microb. Biotechnol..

[27] Deist B.R., Rausch M.A., Fernandez-Luna M.T., Adang M.J., Bonning B.C. (2014). Bt toxin modification for enhanced efficacy. Toxins.

[28] Pardo-López L., Muñoz-Garay C., Porta H., Rodríguez-Almazán C., Soberón M., Bravo A. (2009). Strategies to improve the insecticidal activity of Cry toxins from *Bacillus thuringiensis*. Peptides.

[29] Khasdan V., Sapojnik M., Zaritsky A., Horowitz A.R., Boussiba S., Rippa M., Manasherob R., Ben-Dov E. (2007). Larvicidal activities against agricultural pests of transgenic *Escherichia coli* expressing combinations of four genes from *Bacillus thuringiensis*. Arch. Microbiol..

[30] Elleuch J., Jaoua S., Ginibre C., Chandre F., Tounsi S., Zghal R.Z. (2016). Toxin stability improvement and toxicity increase against dipteran and lepidopteran larvae of *Bacillus thuringiensis* crystal protein Cry2Aa. Pest Manag. Sci..

[31] Hu S.B., Liu P., Ding X.Z., Yan L., Sun Y.J., Zhang Y.M., Li W.P., Xia L.Q. (2009). Efficient constitutive expression of chitinase in the mother cell of *Bacillus thuringiensis* and its potential to enhance the toxicity of Cry1Ac protoxin. Appl. Microbiol. Biotechnol..

[32] Leetachewa S., Khomkhum N., Sakdee S., Wang P., Moonsom S. (2018). Enhancement of insect susceptibility and larvicidal efficacy of Cry4Ba toxin by calcofluor. Parasites Vectors.

[33] Pan X., Xu Z., Li L., Shao E., Chen S., Huang T., Chen Z., Rao W., Huang T., Zhang L. (2017). Adsorption of Insecticidal Crystal Protein Cry11Aa onto Nano-Mg(OH)2: Effects on bioactivity and anti-ultraviolet ability. J. Agric. Food Chem..

[34] Pérez C., Fernández L.E., Sun J., Folch J.L., Gill S.S., Soberón M., Bravo A. (2005). *Bacillus thuringiensis* subsp. israelensis Cyt1Aa synergizes Cry11Aa toxin by functioning as a membrane-bound receptor. Proc. Natl. Acad. Sci. USA.

[35] Cantón P.E., Reyes E.Z., De Escudero I.R., Bravo A., Soberón M. (2011). Binding of *Bacillus thuringiensis* subsp. israelensis Cry4Ba to Cyt1Aa has an important role in synergism. Peptides.

[36] Park H.W., Pino B.C., Kozervanich-Chong S., Hafkenscheid E.A., Oliverio R.M., Federici B.A., Bideshi D.K. (2013). Cyt1Aa from *Bacillus thuringiensis* subsp. israelensis enhances mosquitocidal activity of B. thuringiensis subsp. kurstaki HD-1 against Aedes aegypti but not Culex quinquefasciatus. J. Microbiol. Biotechnol..

[37] Elleuch J., Zghal R.Z., Jemaà M., Azzouz H., Tounsi S., Jaoua S. (2014). New *Bacillus thuringiensis* toxin combinations for biological control of lepidopteran larvae. Int. J. Biol. Macromol..

[38] Yang J., Quan Y., Sivaprasath P., Shabbir M.Z., Wang Z., Ferré J., He K. (2018). Insecticidal activity and synergistic combinations of ten different Bt toxins against *Mythimna separata* (Walker). Toxins.

[39] Wirth M.C., Jiannino J.A., Federici B.A., Walton W.E. (2004). Synergy between toxins of *Bacillus thuringiensis* subsp. israelensis and Bacillus sphaericus. J. Med. Entomol..

[40] Luo X., Chen L., Huang Q., Zheng J., Zhou W., Peng D., Ruan L., Sun M. (2013). *Bacillus thuringiensis* metalloproteinase Bmp1 functions as a nematicidal virulence factor. Appl. Environ. Microbiol..

[41] Matsumoto R., Shimizu Y., Howlader M.T.H., Namba M., Iwamoto A., Sakai H., Hayakawa T. (2014). Potency of insect-specific scorpion toxins on mosquito control using *Bacillus thuringiensis* Cry4Aa. J. Biosci. Bioeng..

[42] García-Gómez B.I., Cano S.N., Zagal E.E., Dantán-Gonzalez E., Bravo A., Soberón M. (2019). Insect Hsp90 chaperone assists *Bacillus thuringiensis* Cry toxicity by enhancing protoxin binding to the receptor and by protecting protoxin from gut protease degradation. mBio.

[43] El-Menofy W., Osman G., Assaeedi A., Salama M. (2014). A novel recombinant baculovirus overexpressing a *Bacillus thuringiensis* Cry1Ab toxin enhances insecticidal activity. Biol. Proced. Online.

[44] Xia L., Long X., Ding X., Zhang Y. (2009). Increase in insecticidal toxicity by fusion of the *cry1Ac* gene from *Bacillus thuringiensis* with the neurotoxin gene *hwtx-I*. Curr. Microbiol..

[45] Li W.P., Xia L.Q., Ding X.Z., Lv Y., Luo Y.S., Hu S.B., Yin J., Yan F. (2012). Expression and characterization of a recombinant Cry1Ac crystal protein fused with an insect-specific neurotoxin *ω-ACTX-Hv1a* in *Bacillus thuringiensis*. Gene.

[46] Sun Y., Fu Z., He X., Yuan C., Ding X., Xia L. (2016). Enhancement of *Bacillus thuringiensis* insecticidal activity by combining Cry1Ac and bi-functional toxin HWTX-XI from spider. J. Invertebr. Pathol..

[47] Sellami S., Jemli S., Abdelmalek N., Cherif M., Abdelkefi-Mesrati L., Tounsi S., Jamoussi K. (2018). A novel Vip3Aa16-Cry1Ac chimera toxin: Enhancement of toxicity against *Ephestia kuehniella*, structural study and molecular docking. Int. J. Biol. Macromol..

[48] Hu X., Liu Z., Li Y., Ding X., Xia L., Hu S. (2014). PirB-Cry2Aa hybrid protein exhibits enhanced insecticidal activity against *Spodoptera exigua* larvae. J. Invertebr. Pathol..

[49] Tajne S., Sanam R., Gundla R., Gandhi N.S., Mancera R.L., Boddupally D., Vudem D.R., Khareedu V.R. (2012). Molecular modeling of Bt Cry1Ac (DI-DII)-ASAL (*Allium sativum* lectin)-fusion protein and its interaction with aminopeptidase N (APN) receptor of *Manduca sexta*. J. Mol. Graph. Model..

[50] Tajne S., Boddupally D., Sadumpati V., Vudem D.R., Khareedu V.R. (2013). Synthetic fusion-protein containing domains of Bt Cry1Ac and *Allium sativum* lectin (ASAL) conferred enhanced insecticidal activity against major lepidopteran pests. J. Biotechnol..

[51] Guo C.H., Zhao S.T., Ma Y., Hu J.J., Han X.J., Chen J., Lu M.Z. (2012). *Bacillus thuringiensis* Cry3Aa fused to a cellulase-binding peptide shows increased toxicity against the longhorned beetle. Appl. Microbiol. Biotechnol..

[52] Hou J., Cong R., Izumi-Willcoxon M., Ali H., Zheng Y., Bermudez E., McDonald M., Nelson M., Yamamoto T. (2019). Engineering of *Bacillus thuringiensis* Cry proteins to enhance the activity against western corn rootworm. Toxins.

[53] Zhang Y., Zhao D., Yan X., Guo W., Bao Y., Wang W., Wang X. (2017). Identification and characterization of *Hyphantria cunea* aminopeptidase N as a binding protein of *Bacillus thuringiensis* Cry1Ab35 toxin. Int. J. Mol. Sci..

[54] Park Y., Abdullah M.A.F., Taylor M.D., Rahman K., Adang M.J. (2009). Enhancement of *Bacillus thuringiensis* Cry3Aa and Cry3Bb toxicities to coleopteran larvae by a toxin-binding fragment of an insect cadherin. Appl. Environ. Microbiol..

[55] Peng D., Xu X., Ruan L., Yu Z., Sun M. (2010). Enhancing Cry1Ac toxicity by expression of the *Helicoverpa armigera* cadherin fragment in *Bacillus thuringiensis*. Res. Microbiol..

[56] Gao Y., Jurat-Fuentes J.L., Oppert B., Fabrick J.A., Liu C., Gao J., Lei Z. (2011). Increased toxicity of *Bacillus thuringiensis* Cry3Aa against *Crioceris quatuordecimpunctata*, *Phaedon brassicae* and *Colaphellus bowringi* by a *Tenebrio molitor* cadherin fragment. Pest Manag. Sci..

[57] Rahman K., Abdullah M.A.F., Ambati S., Taylor M.D., Adang M.J. (2012). Differential protection of Cry1Fa toxin against *Spodoptera frugiperda* larval gut proteases by cadherin orthologs correlates with increased synergism. Appl. Environ. Microbiol..

[58] Park Y., Hua G., Taylor M.D., Adang M.J. (2014). A coleopteran cadherin fragment synergizes toxicity of *Bacillus thuringiensis* toxins Cry3Aa, Cry3Bb, and Cry8Ca against lesser mealworm, *Alphitobius diaperinus* (Coleoptera: Tenebrionidae). J. Invertebr. Pathol..

[59] Park Y., Hua G., Ambati S., Taylor M., Adang M.J. (2019). Binding and synergizing motif within coleopteran cadherin enhances Cry3Bb toxicity on the Colorado Potato Beetle and the lesser mealworm. Toxins.

[60] Schnepf H.E., Whiteley H.R. (1981). Cloning and expression of the *Bacillus thuringiensis* crystal protein gene in *Escherichia coli*. Proc. Natl. Acad. Sci. USA.

[61] Schnepf H.E., Wongs H.C., Whiteley5 H.R. (1985). The amino acid sequence of a crystal protein from *Bacillus thuringiensis* deduced from the DNA base sequence. J. Biol. Chem..

[62] Wong S., Wilcox E., Edwards D., Herrnstadt C. (1986). Process for Altering the Host Range of *Bacillus thuringiensis* Toxins, and Novel Toxins Produced Thereby 1986. Pattent Application No. CA19860617139.

[63] Smith H.O., Wilcox K.W. (1970). A restriction enzyme from *Hemophilus influenzae* I. Purification and general properties. J. Mol. Biol..

[64] Sanger F., Coulson A. (1975). A rapid method for determining sequences in DNA by primed synthesis with DNA polymerase. J. Mol. Biol..

[65] Sanger F., Nicklen S., Coulson A.R. (1977). DNA sequencing with chain-terminating inhibitors. Proc. Natl. Acad. Sci. USA.

[66] Shortle D., Botstein D. (1982). Single-stranded gaps as localized targets for in vitro mutagenesis. Basic Life Sci..

[67] Myers R., Lerman L., Maniatis T. (1985). A general method for saturation mutagenesis of cloned DNA fragments. Science.

[68] Jellis C., Bass And D., Beerman N., Dennis C., Farrell K., Piot J.C., Rusche J., Carson H., Witt D. (1989). Molecular biology of *Bacillus thuringiensis* and potential benefits to agriculture. Isr. J. Entomol..

[69] Saiki R., Gelfand D., Stoffel S., Scharf S., Higuchi R., Horn G., Mullis K., Erlich H. (1988). Primer-directed enzymatic amplification of DNA with a thermostable DNA polymerase. Science.

[70] Ge A.Z., Shivarova N.I., Deant D.H. (1989). Location of the *Bombyx mori* specificity domain on a *Bacillus thuringiensis* 6-endotoxin protein. Proc. Nat. Acad. Sci. USA.

[71] Höfte H., Whiteley H. (1989). Insecticidal crystal proteins of *Bacillus thuringiensis*. Microbiol. Rev..

[72] Schnepf H.E., Tomczak K., Ortega J.P., Whiteleys H.R. (1990). Specificity-determining regions of a Lepidopteran-specific insecticidal protein produced by *Bacillus thuringiensis*. J. Biol. Chem..

[73] Widner W.R., Whiteley H.R. (1990). Location of the Dipteran specificity region in a Lepidopteran-Dipteran crystal protein from *Bacillus thuringiensis*. J. Bacteriol..

[74] Ge A.Z., Rivers D., Milne R., Dean D.H. (1991). Functional domains of *Bacillus thuringiensis* insecticidal crystal proteins. J. Biol. Chem..

[75] Weber H., Weissmann C. (1983). Formation of genes coding for hybrid proteins by recoinbination between related, cloned genes in *Escherichia coli*. Nucl. Acids Res..

[76] Caramori T., Albertini A.M., Galizzi A. (1991). In vivo generation of hybrids between two *Bacillus thuringiensis* insect-toxin-encoding genes. Gene.

[77] Bosch D., Schipper B., Van Der Kleij H., De Maagd R.A., Stiekema W.J. (1994). Recombinant *Bacillus thuringiensis* crystal proteins with new properties: Possibilities for resistance management. Biotechnology.

[78] De Maagd R.A., Kwa M.S.G., Van Der Klei H., Yamamoto T., Schipper B., Vlak J.M., Stiekema W.J., Bosch D. (1996). Domain III substitution in *Bacillus thuringiensis* delta-endotoxin CryIA(b) results in superior toxicity for *Spodoptera exigua* and altered membrane protein recognition. Appl. Environ. Microbiol..

[79] De Maagd R.A., Weemen-Hendriks M., Stiekema W., Bosch D. (2000). *Bacillus thuringiensis* delta-endotoxin Cry1C domain III can function as a specificity determinant for *Spodoptera exigua* in different, but not all, Cry1-Cry1C hybrids. Appl. Environ. Microbiol..

[80] Karlova R., Weemen-Hendriks M., Naimov S., Ceron J., Dukiandjiev S., De Maagd R.A. (2005). *Bacillus thuringiensis* δ-endotoxin Cry1Ac domain III enhances activity against *Heliothis virescens* in some, but not all Cry1-Cry1Ac hybrids. J. Invertebr. Pathol..

[81] Sanchis V., Gohar M., Chaufaux J., Arantes O., Meier A., Agaisse H., Cayley J., Lereclus D. (1999). Development and field performance of a broad-spectrum nonviable asporogenic recombinant strain of *Bacillus thuringiensis* with greater potency and UV resistance. Appl. Environ. Microbiol..

[82] Naimov S., Weemen-Hendriks M., Dukiandjiev S., De Maagd R.A. (2001). *Bacillus thuringiensis* delta-endotoxin Cry1 hybrid proteins with increased activity against the Colorado potato beetle. Appl. Environ. Microbiol..

[83] Walters F.S., Defontes C.M., Hart H., Warren G.W., Chen J.S. (2010). Lepidopteran-active variable-region sequence imparts coleopteran activity in eCry3.1Ab, an engineered *Bacillus thuringiensis* hybrid insecticidal protein. Appl. Environ. Microbiol..

[84] Walters F.S., Stacy C.M., Mi K.L., Palekar N., Chen J.S. (2008). An engineered chymotrypsin/cathepsin G site in domain I renders *Bacillus thuringiensis* Cry3A active against western corn rootworm larvae. Appl. Environ. Microbiol..

[85] Shah J.V., Yadav R., Ingle S.S. (2017). Engineered Cry1Ac-Cry9Aa hybrid *Bacillus thuringiensis* delta-endotoxin with improved insecticidal activity against *Helicoverpa armigera*. Arch. Microbiol..

[86] Dulau L., Cheyrou A., Aigle M. (1989). Directed mutagenesis using PCR. Nucleic Acids Res..

[87] Wu D., Aronson A.I. (1992). Localized mutagenesis defines regions of the *Bacillus thuringiensis* delta-endotoxin involved in toxicity and specificity. J. Biol. Chem..

[88] Padilla C., Pardo-López L., De La Riva G., Gómez I., Sánchez J., Hernandez G., Nuñez M.E., Carey M.P., Dean D.H., Alzate O. (2006). Role of tryptophan residues in toxicity of Cry1Ab toxin from *Bacillus thuringiensis*. Appl. Environ. Microbiol..

[89] Alzate O., Osorio C., Florez A.M., Dean D.H. (2010). Participation of valine 171 in α-helix 5 of *Bacillus thuringiensis* Cry1Ab δ-endotoxin in translocation of toxin into *Lymantria dispar* midgut membranes. Appl. Environ. Microbiol..

[90] Smedley D.P., Ellar D.J. (1996). Mutagenesis of three surface-exposed loops of a *Bacillus thuringiensis* insecticidal toxin reveals residues important for toxicity, receptor recognition and possibly membrane insertion. Microbiology.

[91] Wu S.J., Dean D.H. (1996). Functional significance of loops in the receptor binding domain of *Bacillus thuringiensis* CryIIIA delta-endotoxin. J. Mol. Biol..

[92] Rajamohan F., Alzate O., Cotrill J.A., Curtiss A., Dean D.H. (1996). Protein engineering of *Bacillus thuringiensis* endotoxin: Mutations at domain II of CryIAb enhance receptor affinity and toxicity toward gypsy moth larvae. Proc. Natl. Acad. Sci. USA.

[93] Wu S.-J., Koller C.N., Miller D.L., Bauer L.S., Dean D.H. (2000). Enhanced toxicity of *Bacillus thuringiensis* Cry3A N-endotoxin in coleopterans by mutagenesis in a receptor binding loop. FEBS Lett..

[94] Mcneil B.C., Dean D.H. (2011). *Bacillus thuringiensis* Cry2Ab is active on *Anopheles* mosquitoes: Single D block exchanges reveal critical residues involved in activity. FEMS Microbiol. Lett..

[95] Nicholls C.N., Ahmad W., Ellar D.J. (1989). Evidence for two different types of insecticidal P2 toxins with dual specificity in *Bacillus thuringiensis* subspecies. J. Bacteriol..

[96] Lv Y., Tang Y., Zhang Y., Xia L., Wang F., Ding X., Yi S., Li W., Yin J. (2011). The role of β20-β21 loop structure in insecticidal activity of Cry1Ac toxin from *Bacillus thuringiensis*. Curr. Microbiol..

[97] Gómez I., Ocelotl J., Sánchez J., Lima C., Martins E., Rosales-Juárez A., Aguilar-Medel S., Abad A., Dong H., Monnerat R. (2018). Enhancement of *Bacillus thuringiensis* Cry1Ab and Cry1Fa toxicity to *Spodoptera frugiperda* by domain III mutations indicates there are two limiting steps in toxicity as defined by receptor binding and protein stability. Appl. Environ. Microbiol..

[98] Gómez I., Rodríguez-Chamorro D.E., Flores-Ramírez G., Grande R., Zúñiga F., Portugal F.J., Sánchez J., Pacheco S., Bravo A., Soberón M. (2018). *Spodoptera frugiperda* (J. E. Smith) aminopeptidase N1 is a functional receptor of the *Bacillus thuringiensis* Cry1Ca toxin. Appl. Environ. Microbiol..

[99] Xia L., Wang F., Ding X., Zhao X., Fu Z., Quan M., Yu Z. (2008). The role of β18–β19 lobop structure in insecticidal activity of Cry1Ac toxin from *Bacillus thuringiensis*. Chin. Sci. Bull..

[100] Xiang W.F., Qiu X.L., Zhi D.X., Min Z.X., Yuan L., Quan Y.Z. (2009). N546 in β18-β19 loop is important for binding and toxicity of the *Bacillus thuringiensis* Cry1Ac toxin. J. Invertebr. Pathol..

[101] Wang F., Liu Y., Zhang F., Chai L., Ruan L., Peng D., Sun M. (2012). Improvement of crystal solubility and increasing toxicity against *Caenorhabditis elegans* by asparagine substitution in block 3 of *Bacillus thuringiensis* crystal protein Cry5Ba. Appl. Environ. Microbiol..

[102] Angsuthanasombat C., Crickmore N., Ellar D.J. (1993). Effects on toxicity of eliminating a cleavage site in a predicted interhelical loop in *Bacillus thuringiensis* CrylVB 6-endotoxin. FEMS Microbiol. Lett..

[103] Abdullah M.A.F., Alzate O., Mohammad M., McNall R.J., Adang M.J., Dean D.H. (2003). Introduction of *Culex* toxicity into *Bacillus thuringiensis* Cry4Ba by protein engineering. Appl. Environ. Microbiol..

[104] Abdullah M.A.F., Dean D.H. (2004). Enhancement of Cry19Aa mosquitocidal activity against *Aedes aegypti* by mutations in the putative loop regions of domain II. Appl. Environ. Microbiol..

[105] Liu X.S., Dean D.H. (2006). Redesigning *Bacillus thuringiensis* Cry1Aa toxin into a mosquito toxin. Protein Eng. Des. Sel..

[106] Zhou Z., Liu Y., Liang G., Huang Y., Bravo A., Soberón M., Song F., Zhou X., Zhang J. (2017). Insecticidal specificity of Cry1Ah to *Helicoverpa armigera* is determined by binding of APN1 via domain II loops 2 and 3. Appl. Environ. Microbiol..

[107] Mandal C.C., Gayen S., Basu A., Ghosh K.S., Dasgupta S., Maiti M.K., Sen S.K. (2007). Prediction-based protein engineering of domain I of Cry2A entomocidal toxin of *Bacillus thuringiensis* for the enhancement of toxicity against lepidopteran insects. Protein Eng. Des. Sel..

[108] Smith G.P., Ellar D.J. (1994). Mutagenesis of two surface-exposed loops of the *Bacillus thuringiensis* CrylC δ-endotoxin affects insecticidal specificity. Biochem. J..

[109] Ner S.S., Goodin D.B., Smith M. (1988). A simple and efficient procedure for generating random point mutations and for codon replacements using mixed oligodeoxynucleotides. DNA.

[110] Kumar A.S.M., Aronson A.I. (1999). Analysis of mutations in the pore-forming region essential for insecticidal activity of a *Bacillus thuringiensis* delta-endotoxin. J. Bacteriol..

[111] Leung D., Chen E., Goeddel D. (1989). A method for random mutagenesis of a defined DNA segment using a modified polymerase chain reaction. Technique.

[112] Van Dillewijn P., Vilchez S., Paz J.A., Ramos J.L. (2004). Plant-dependent active biological containment system for recombinant rhizobacteria. Environ. Microbiol..

[113] Shu C., Liu R., Wang R., Zhang J., Feng S., Huang D., Song F. (2007). Improving toxicity of *Bacillus thuringiensis* strain contains the *cry8Ca* gene specific to *Anomala corpulenta* larvae. Curr. Microbiol..

[114] Shan S., Zhang Y., Ding X., Hu S., Sun Y., Yu Z., Liu S., Zhu Z., Xia L. (2011). A Cry1ac toxin variant generated by directed evolution has enhanced toxicity against lepidopteran insects. Curr. Microbiol..

[115] Stemmer W. (1994). Rapid evolution of a protein in vitro by DNA shuffling. Nature.

[116] Stemmer W.P.C. (1994). DNA shuffling by random fragmentation and reassembly: In vitro recombination for molecular evolution. Proc. Natl. Acad. Sci. USA..

[117] Knight J.S., Broadwell A.H., Grant W.N., Shoemaker C.B. (2004). A strategy for shuffling numerous *Bacillus thuringiensis* crystal protein domains. J. Econ. Entomol..

[118] Florez A.M., Suarez-Barrera M.O., Morales G.M., Rivera K.V., Orduz S., Ochoa R., Guerra D., Muskus C. (2018). Toxic activity, molecular modeling and docking simulations of *Bacillus thuringiensis* Cry11 toxin variants obtained via DNA shuffling. Front. Microbiol..

[119] Shu C., Zhou J., Crickmore N., Li X., Song F., Liang G., He K., Huang D., Zhang J. (2016). In vitro template-change PCR to create single crossover libraries: A case study with *Bacillus thuringiensis* Cry2A toxins. Sci. Rep..

[120] Zhao H., Giver L., Shao Z., Affholter J.A., Arnold F.H. (1998). Molecular evolution by staggered extension process (StEP) in vitro recombination. Nat. Biotechnol..

[121] Zhang Y., Buchholz F., Muyrers J.P.P., Stewart A.F. (1998). A new logic for DNA engineering using recombination in *Escherichia coli*. Nat. Genet..

[122] Zhang Y., Muyrers J., Testa G., Stewart A. (2000). DNA cloning by homologous recombination in *Escherichia coli*. Nat. Biotechnol..

[123] Smith G. (1985). Filamentous fusion phage: Novel expression vectors that display cloned antigens on the virion surface. Science.

[124] De La Cruz V.F., Lal A.A., McCutchan T.F. (1988). Immumogenicity and epitope mapping of foreign sequences via genetically engineered filamentous phage. J. Biol. Chem..

[125] Scott J., Smith G. (1990). Searching for peptide ligands with an epitope library. Science.

[126] Landon L., Deutscher S. (2003). Combinatorial discovery of tumor targeting peptides using phage display. J. Cell. Biochem..

[127] Luzar J., Štrukelj B., Lunder M. (2016). Phage display peptide libraries in molecular allergology: From epitope mapping to mimotope-based immunotherapy. Eur. J. Allergy Clin. Immunol..

[128] Marzari R., Edomi P., Bhatnagar R.K., Ahmad S., Selvapandiyan A., Bradbury A. (1997). Phage display of *Bacillus thuringiensis* CryIA(a) insecticidal toxin. FEBS Lett..

[129] Kasman L.M., Lukowiak A.A., Garczynski S.F., Mcnall R.J., Youngman P., Adang M.J. (1998). Phage display of a biologically active *Bacillus thuringiensis* toxin. Appl. Environ. Microbiol..

[130] Vilchez S., Jacoby J., Ellar D.J. (2004). Display of biologically functional insecticidal toxin on the surface of lambda phage. Appl. Environ. Microbiol..

[131] Pacheco S., Gómez I., Sato R., Bravo A., Soberón M. (2006). Functional display of *Bacillus thuringiensis* Cry1Ac toxin on T7 phage. J. Invertebr. Pathol..

[132] Pacheco S., Cantón E., Zuñiga-Navarrete F., Pecorari F., Bravo A., Soberón M. (2015). Improvement and efficient display of *Bacillus thuringiensis* toxins on M13 phages and ribosomes. AMB Express.

[133] Ishikawa H., Hoshino Y., Motoki Y., Kawahara T., Kitajima M., Kitami M., Watanabe A., Bravo A., Soberon M., Honda A. (2007). A system for the directed evolution of the insecticidal protein from *Bacillus thuringiensis*. Mol. Biotechnol..

[134] Fujii Y., Tanaka S., Otsuki M., Hoshino Y., Morimoto C., Kotani T., Harashima Y., Endo H., Yoshizawa Y., Sato R. (2013). Cry1Aa binding to the cadherin receptor does not require conserved amino acid sequences in the domain II loops. Biosci. Rep..

[135] Endo H., Kobayashi Y., Hoshino Y., Tanaka S., Kikuta S., Tabunoki H., Sato R. (2014). Affinity maturation of Cry1Aa toxin to the *Bombyx mori* cadherin-like receptor by directed evolution based on phage display and biopanning selections of domain II loop 2 mutant toxins. Microbiologyopen.

[136] Craveiro K.I.C., Gomes J.E., Silva M.C.M., Macedo L.L.P., Lucena W.A., Silva M.S., de Souza J.D.A., Oliveira G.R., Quezado de Magalhães M.T., Santiago A.D. (2010). Variant Cry1Ia toxins generated by DNA shuffling are active against sugarcane giant borer. J. Biotechnol..

[137] Oliveira G.R., Silva M.C., Lucena W.A., Nakasu E.Y., Firmino A.A., Beneventi M.A., Souza D.S., Gomes J.E., Da De Souza J., Rigden D.J. (2011). Improving Cry8Ka toxin activity towards the cotton boll weevil (*Anthonomus grandis*). BMC Biotechnol..

[138] Pigott C.R., King M.S., Ellar D.J. (2008). Investigating the properties of *Bacillus thuringiensis* Cry proteins with novel loop replacements created using combinatorial molecular biology. Appl. Environ. Microbiol..

[139] Andris-Widhopf J., Rader C., Steinberger P., Barbas C., Fuller R. (2000). Methods for the generation of chicken monoclonal antibody fragments by phage display. J. Immunol. Methods.

[140] Domínguez-Flores T., Romero-Bosquet M.D., Gantiva-Díaz D.M., Luque-Navas M.J., Berry C., Osuna A., Vílchez S. (2017). Using phage display technology to obtain Crybodies active against non-target insects. Sci. Rep..

[141] Shao E., Lin L., Chen C., Chen H., Zhuang H., Wu S., Sha L., Guan X., Huang Z. (2016). Loop replacements with gut-binding peptides in Cry1Ab domain II enhanced toxicity against the brown planthopper, *Nilaparvata lugens* (Stål). Sci. Rep..

[142] Esvelt K.M., Carlson J.C., Liu D.R. (2011). A system for the continuous directed evolution of biomolecules. Nature.

[143] Leconte A.M., Dickinson B.C., Yang D.D., Chen I.A., Allen B., Liu D.R. (2013). A population-based experimental model for protein evolution: Effects of mutation rate and selection stringency on evolutionary outcomes. Biochemistry.

[144] Dickinson B.C., Packer M.S., Badran A.H., Liu D.R. (2014). A system for the continuous directed evolution of proteases rapidly reveals drug-resistance mutations. Nat. Commun..

[145] Hubbard B.P., Badran A.H., Zuris J.A., Guilinger J.P., Davis K.M., Chen L., Tsai S.Q., Sander J.D., Joung J.K., Liu D.R. (2015). Continuous directed evolution of DNA-binding proteins to improve TALEN specificity. Nat. Methods.

[146] Badran A.H., Guzov V.M., Huai Q., Kemp M.M., Vishwanath P., Kain W., Nance A.M., Evdokimov A., Moshiri F., Turner K.H. (2016). Continuous evolution of *Bacillus thuringiensis* toxins overcomes insect resistance. Nature.

[147] Baxter S.W., Badenes-Pérez F.R., Morrison A., Vogel H., Crickmore N., Kain W., Wang P., Heckel D.G., Jiggins C.D. (2011). Parallel evolution of *Bacillus thuringiensis* toxin resistance in Lepidoptera. Genetics.

[148] Tiewsiri K., Wang P. (2011). Differential alteration of two aminopeptidases N associated with resistance to *Bacillus thuringiensis* toxin Cry1Ac in cabbage looper. Proc. Natl. Acad. Sci. USA.

