# Supplementary material for: Making 3D-Cry Toxin Mutants: Much More Than a Tool of Understanding Toxins Mechanism of Action
Source: Toxins (Basel). 2020 Sep 16;12(9):600. doi: 10.3390/toxins12090600 (PMC7551160; doi:10.3390/toxins12090600)
Supplement: Supplementary file 1 [file toxins-12-00600-s001.pdf]

Supplementary Materials: Making 3D-Cry Toxin Mutants: Much More Than a Tool of Understanding Toxins Mechanism of Action

Susana Vílchez

Table S1. Sequence of some mutant toxins mentioned in the review. Parental toxin, mutant name and sequence, and domain evolved are indicated. Single mutants, which names are the same as the mutation introduced, are excluded.

Parental Toxin Evolved	Mutant Name and Sequence	Domain Evolved[1]	Reference
CryIA(b)	**P26-3:** A119T, M130I, G201D<br>**P48a14:** E101K, E116K, R217H<br>**P48c5:** E116K, A187T<br>**P36a65:** T122I, A125V<br>**P95a76:** N123Y<br>**P95a86:** T188S<br>**P98c1:** T188S<br>**P99c62:** N4Y, N105Y<br>**P107c22:** N94K, N194K<br>**107c25:** F184I<br>**114a30:** Q95K	DI*	[68]
ICPC73	**OSU 4205:**<br>ICPC73 toxin with a ICPA1 fragment from Domain II.	DII*	[70]
CryIE	**G27:**<br>CryIE (DIDII)-CryIC (DIII)	DIII	[77]
CryIA(b)	**H04:**<br>CryIA(b) (DIDII)-CryIC (DIII)	DIII	[78]
CryIIIA	**Triple mutant:**<br>S484A, R485A, G486A	DII (Loop 3)	[91]
Cry1Ab	**DF-1:** Triple mutant<br>N372A, A282G, L283S	DII	[92]
Cry1C	**Cry1C/Ab hybrid:**<br>DI-DIV form Cry1C and DIV-DVII from Cry1Ab	DI-DVII	[81]
Cry1Ba	**BBC13:** Cry1Ba (DIDII)-CryIC (DIII)<br><br>**BBC15:** Cry1Ba (DIDII)-CryIC (DIII)	DIII	[79]
Cry1Fa	**FFC1:** Cry1Fa (DIDII)-CryIC (DIII)	DIII	[79]
Cry3A	**A1:**<br>L1:R345A,Y350F,Y351F	DII	[93]
Cry3A	**A2:**<br>L1:R345A, Y350, Y351	DII	[93]

Cry1Ia	**1Ia/1Ia/1Ba hybrid:**<br>Cry1Ia (DIDII)-Cry1Ba (DIII)	DI, DII, DIII	[82]
Cry1Ba	**1Ba/1Ia/1Ba hybrid:**<br>Cry1Ba (DI)-Cry1Ba (DIIDIII)	DI, DII, DIII	[82]
Cry4Ba using Loop3 from Cry4Aa	**4BL3PAT:**<br>L3:^{452}VIPATYNS^{459}	DII	[103]
Cry19Aa using loop from Cry4Ba	**19AL1L2:**<br>L1: ^{355}YQDLR^{359}<br>L2: deleted	DII	[104]
Cry1Ca and Cry1Fb using DIII of Cry1Ac	**RK15:** Cry1Ca (DIDII)-Cry1Ac (DIII)<br>**RK12:** Cry1Fb (DIDII)-Cry1Ac (DIII)		[80]
Cry1Aa using loop 1 from Cry4Ba	**1AaMosq**<br>L1: ^{311}YQDL^{314}<br>L2:^{365}GSSPG^{369}	DII	[105]
Cry8Ca2	**M100:** E642G<br>**M102:** Q439P	DIII<br>DII	[113]
Cry2A	**D42:**<br>Deleted first 42 aa (αH1)	DI	[107]
Cry1Aa1	**R5-51:**<br>**L2:**^{261}LSSPLYRR**KSALPQV**NNQELFVLD^{384}	DII (loop 2)	[133]
Cry3A	**mCry3A:**<br>^{153}NPAAPFRN^{160}<br><br>Introduction of a Chimotripsin/catepsina site	DI<br>(Loop α-helix 3 and 4)	[84]
Cry1Ia12synth	**Variant 1:**<br>D233N (DI), E639G (DIII)<br>**Variant 2:**<br>D233N (DI)<br>**Variant 3:**<br>I116T (DI), L266F (DI),<br>K580R (DIII)<br>**Variant 4:**<br>M45V (N-terminus), D233N (DI)	DI, DII, DIII	[136]
mCry3A	**eCry3.1Ab:**<br>(GenBank GU327680)	DIII	[83]
Cry8Ka1	**Cry8Ka5:**<br>R82Q, Y260C, P321A, R508G, K538E, E594N	DI, DII, DIII	[137]
Cry2Ad	**R24:**<br>Recombination at position ^{416}NY^{417}	Recombination at DII	[119]

	**R26:**<br>Recombination at position ^{440}RPL^{442}<br><br>**R27:**<br>Recombination at position ^{455}GTPGGA^{460}		
Cry1Ab	**L1-P2S:** ^{278}CLMSSQAAC^{286}<br>**L2-P2S:** ^{335}CLMSSQAAC^{343}<br>**L3-P2S:** ^{401}CLMSSQAAC^{409}<br>**L1-P1Z:** ^{278}CHLPRLPQC^{286}<br>**L2-P1Z:** ^{335}CHLPRLPQC^{343}	DII	[141]
Cry1Ac	**A01s:** Not available<br>**C04s:** Not available<br>**C05s:** Not available	Not available	[146]
Cry1Ai	**Cry1Ai-h-loop2:**<br>^{390}RPFNIGINNQQ^{400}<br>**Cry1Ai-h-loop2&3:**<br>^{390}RPFNIGINNQQ^{400}<br>^{455}SMFRSGSSSSVSIIR^{469}	DII	[106]
Cry9Aa	**Cry1Ac-Cry9Aa:**<br>Cry1Aa (DI)-Cry9Aa (DIIDIII)<br>**Cry1Ac-Cry9AaMod:**<br>Helix 1 removed	DI, DII, DIII	[85]
Chimeric protein Cry4Ba and Cry1Ac	**Cry(4Ba-1Ac):**<br>Cry4Ba (DI-DIV)-Cry1Ac (DV-DVII)	DI-DVII	[21]
Cry1Aa13	**Cry1Aa13-A8:**<br>L2: ^{367}GAREGSSSAYDYW^{379}<br>**Cry1Aa13-A12:**<br>L2:^{367}GARGDPDFDHSTSYYLDYC^{385}	DII	[140]
Cry11Aa, Cry11Ba, and Cry11Bb	**Variant 8:**<br>(Gene bank MH068787)<br>73 aa deletion at N-terminal end (Domain I) and 13 aa substitutions at Domain II and III.	DI, DII, DIII	[118]
IP3-1: an artificial mutant derived from Cry3Aa1	**IP3-2:**<br>6 extra mutations: K152E, R158R, I340V, K384E, Q472L, F589L<br>**IP3-3:**<br>8 extra mutations:<br>K152E, R158R, E221S, K222S, I340V, K384E, Q472L, F589L<br>**IP3-4:**<br>7 extra mutations:<br>K63R, N97D, Q119H, K152E, R158E, F584L, M493V	DI, DII, DIII	[52]

**IP3-5:**<br>9 extra mutations:<br>K63R, N97D, Q119H, K152E, R158E, E221S,<br>K222S, F584L, M593V
**IP3-6:**<br>7 extra mutations:<br>K63E, K152E, R158E, Q232H, K496E,<br>K557H, S610T
**IP3-7:**<br>K63E, K152E, R158E, E221S, K222S, Q232H,<br>K496E, K557H, S610T

[1] DI: Domain 1; DII: Domain 2; DIII: Domain 3. Di *: Indicated the domain evolved although it was not known at the time.
